# Effects of intra-articular applied rat BMSCs expressing alpha-calcitonin gene-related peptide or substance P on osteoarthritis pathogenesis in a murine surgical osteoarthritis model

**DOI:** 10.1186/s13287-025-04155-2

**Published:** 2025-03-05

**Authors:** Sabine Stöckl, Shahed Taheri, Verena Maier, Amir Asid, Martina Toelge, Hauke Clausen-Schaumann, Arndt Schilling, Susanne Grässel

**Affiliations:** 1 Department of Orthopaedic Surgery, Experimental Orthopaedics, Centre for Medical Biotechnology, University of Regensburg, ZMB im Biopark, 1 Am Biopark 9, 93053 Regensburg, Germany; 2 Department of Trauma Surgery, Orthopedics and Plastic Surgery, University Medicine Göttingen, Lower Saxony, Germany; 3 Center for Applied Tissue Engineering and Regenerative Medicine (CANTER), University of Applied Sciences Munich, Munich, Germany; 4 Department of Medical Microbiology and Hygiene, University of Regensburg, Regensburg, Germany; 5 Department of Pediatric Hematology, Oncology and Stem Cell Transplantation, University of Regensburg, Regensburg, Germany

**Keywords:** Osteoarthritis, DMM, SP, αCGRP, BMSC, Nanoct, AFM, Serum marker, Motion analysis

## Abstract

**Background:**

About 655 million persons worldwide are affected by osteoarthritis (OA). As no therapy modifies disease progression long-term, there is an immense clinical need for novel therapies. The joints are innervated by alpha calcitonin gene-related peptide (αCGRP)- and substance P (SP)-positive sensory nerve fibers. Both neuropeptides have trophic effects on target cells within the joints. The aim of this study was to examine the effects of SP- and αCGRP-expressing intra-articular (i.a.) applied rat(r)BMSC on cartilage and subchondral bone structural changes after OA induction.

**Methods:**

Mice were subjected to destabilization of the medial meniscus (DMM) surgery, followed by i.a. injections with rBMSC, transduced with lacZ, SP or αCGRP. 2, 8 and 16 weeks after DMM/Sham surgery, motion analysis and serum marker analysis were performed. Cartilage and subchondral bone properties were assessed by OA scoring, atomic force microscopy and nano-CT analysis.

**Results:**

OARSI scores of the medial cartilage compartments indicated induction and progression of OA after DMM surgery in all groups. Differences between the treatment groups were mostly restricted to the lateral cartilage compartments, where αCGRP caused a decrease of structural changes. DMM-rBMSC-αCGRP or -SP mice displayed decreased cartilage stiffness in the cartilage middle zone. DMM-rBMSC-αCGRP mice revealed improved mobility, whereas Sham-rBMSC-SP mice revealed reduced mobility compared to rBMSC-lacZ. With respect to condyle length, subarticular bone and ephiphyseal bone morphology, DMM-rBMSC-SP mice had more alterations indicating either a more progressed OA stage or a more severe OA pathology compared to controls. In addition, DMM-rBMSC-SP mice developed osteophytes already 8 weeks after surgery. Adiponectin serum level was increased in DMM-rBMSC-αCGRP mice, and MIP1b level in DMM-rBMSC-SP mice. Notably, pain and inflammation markers increased over time in rBMSC-SP mice while rBMSC-αCGRP mice revealed a bell-shaped curve with a peak at 8 weeks.

**Conclusions:**

We conclude that i.a. injection of rBMSC in general have a beneficial effect on cartilage matrix structure, subchondral bone microarchitecture and inflammation. rBMSC-αCGRP have anabolic and possible analgesic properties and may attenuate the progression or severity of OA. In contrast, rBMSC-SP exert a more catabolic influence on knee joints of both, Sham and DMM mice, making it a potential candidate for inhibition studies.

**Supplementary Information:**

The online version contains supplementary material available at 10.1186/s13287-025-04155-2.

## Introduction

Osteoarthritis (OA) is one of the most common musculoskeletal disorders worldwide and is an enormous social and economic burden for those affected and for the global health [1]. OA is a disease of the whole joint. Tissues like articular cartilage, subchondral bone, the synovium, adjacent ligaments, the meniscus and even muscles, nerves and local fat pads are prone to impairment in their biology and function [2]. There are various treatment options available for OA, including first line non-pharmacological interventions such as exercise and weight management, and pharmacological interventions such as nonsteroidal anti-inflammatory drugs (NSAIDs) and analgesics. However, the effectiveness of these treatments can vary depending on the stage of the disease, the underlying cause and the individual patient’s response and are only addressing the symptoms mostly without halting or reversing structural tissue damage [3]. Arthroplasty is a gold standard for severe, end stage OA, however to address the clinical need of younger, moderately diseased patients with mild to moderate knee OA, devices as an implantable shock absorber were developed to unload the knee, but the procedure is associated with a long recovery period that limits patient acceptance [4].

Substance P (SP) and alpha calcitonin gene-related peptide (αCGRP) are nociceptive neuropeptides that play crucial roles in pain perception in the periphery and transmission to the central nervous system. Joints are innervated by αCGRP- and SP-positive sensory nerve fibers. Changes in peripheral joint innervation may be partly responsible for degenerative alterations in joint tissues that contribute to the development of OA, to increased pain sensation and to alterations in bone metabolism [5, 6]. In addition to their classical neurological features, trophic effects that are critical for joint tissue and bone homeostasis under physiological and pathophysiological conditions have been reported by our group [7]. Niedermair et al. demonstrated that the absence of SP results in only a slight reduction of the bone resorption rate during murine fracture repair, but concomitantly in a critical reduction of bone formation and mineralization rate [8], whereas the absence of αCGRP leads to increased numbers of dysfunctional mature osteoblasts [9]. Unlike bone, healthy cartilage does neither contain blood vessels nor is it innervated. However, there is evidence that during OA progression, nerves can reach the calcified cartilage layer from the adjacent subchondral bone and with that, SP and αCGRP could also affect chondrocytes within the deep cartilage zone [5, 10]. Suri et al. have even localized SP- and αCGRP-positive nerve fibers in the articular cartilage of OA patients. They hypothesized that during the pathogenesis of OA, fine unmyelinated nerves grow into joint tissues through vascular channels or bone pores, mainly from cartilage adjacent subchondral bone breaching through the tidemark rather than coming from synovium or periosteum [11]. However, so far no SP- or αCGRP positive nerve fibers could be identified transiting through the cartilage adjacent subchondral bone pores from the bone site into the calcified cartilage zone (unpublished data). Of note, vascularization and innervation of the non-calcified articular cartilage zones have been found in a wide range of histological OA stages and are not restricted to end stage OA [11, 12]. In a recent study, we showed that SP and αCGRP affect chondrocytes from healthy donors and from patients with OA differently. In chondrocytes from healthy cartilage, SP had minimal effects compared with its effects on OA chondrocytes, where it induced secretion of inflammatory mediators, inhibited expression of chondrogenic markers and promoted apoptosis and senescence [7]. That is in line with data from our group, showing, that in a murine destabilization-induced knee osteoarthritis model, cartilage degradation was delayed in SP-deficient mice [13]. In vitro treatment with αCGRP on the other hand increased apoptosis and senescence and reduced chondrogenic marker expression in OA chondrocytes, but stimulated an anabolic and protective response in healthy chondrocytes [7]. These in vitro data call for validation in vivo, to understand more precisely the influence of the two neuropeptides during the onset and progression of OA. We therefore studied the appearance of degenerative changes in cartilage and subchondral bone in wild type (WT) mice after surgical destabilization of the medial meniscus (DMM) in the presence of increased concentrations of SP or αCGRP, mediated by intra articular (i.a.) injected rat (r)BMSCs stably transduced with murine SP or αCGRP.

## Materials and methods

### Animals

The DMM model was described as a testosterone-driven pathology, hence male mice were used in this study [14]. Before induction of OA, 8–10 weeks old male C57Bl/6J (WT) mice were purchased from Charles River Laboratories (Sulzfeld, Germany) and were adapted to standard housing conditions under a 12 hour dark/light cycle until the age of 12 weeks. The work has been reported in line with the ARRIVE guidelines 2.0.”

### Destabilization of the medial meniscus (DMM) and intra-articular injection of rBMSC

At an age of 12 weeks, OA was surgically induced following a method described by Glasson [15]. Briefly, after intraperitoneal anesthesia with ketamine (120 mg/kg bodyweight) and xylazin (8 mg/kg bodyweight), a 3 mm skin incision was made between the distal patella and the proximal tibia plateau of the right leg, exposing the knee joint. The joint capsule was opened with a 1–2 mm incision medial to the patellar tendon. For induction of OA, the medial menisco-tibial ligament was dissected carefully after visualization using micro scissors. Sham surgery was performed in the right knee of the OA control group with visualization of the ligament only. Joint capsule and skin were closed and animals received analgesia (buprenorphine in 0,9% NaCl solution, 0,1 mg/g bodyweight). Immediately after surgery, animals were allowed to move freely and recover from surgery. A total of 240 mice were randomly divided into 3 groups: End of experiment at 2 weeks (72 animals), at 8 weeks (96 animals) and at 16 weeks (72 animals) after DMM or sham surgery. In each group, half of the animals were randomly chosen and subjected to DMM surgery, whereas the other half was allocated to sham surgery. Within the DMM and sham groups, respectively, 4 subgroups were formed, depending on the type of intra-articular (i.a.) injection: PBS, rBMSC-lacZ, rBMSC-SP, rBMSC-αCGRP. The PBS and lacZ groups were used as two different controls in this study. PBS treatment served as a control for the effects of an i.a. injection. In contrast, the injection of lacZ-primed rBMSC shall reveal the difference to the neuropeptide-specific effects. The stem cell-specific effect can then be separated from the neuropeptide effects, as lacZ-, SP- and αCGRP- treated animals all received rBMSC injections. Each group consisted of either 9 mice (2 and 16 weeks time point) or 12 mice (8 weeks time point).

**Fig. 1:**
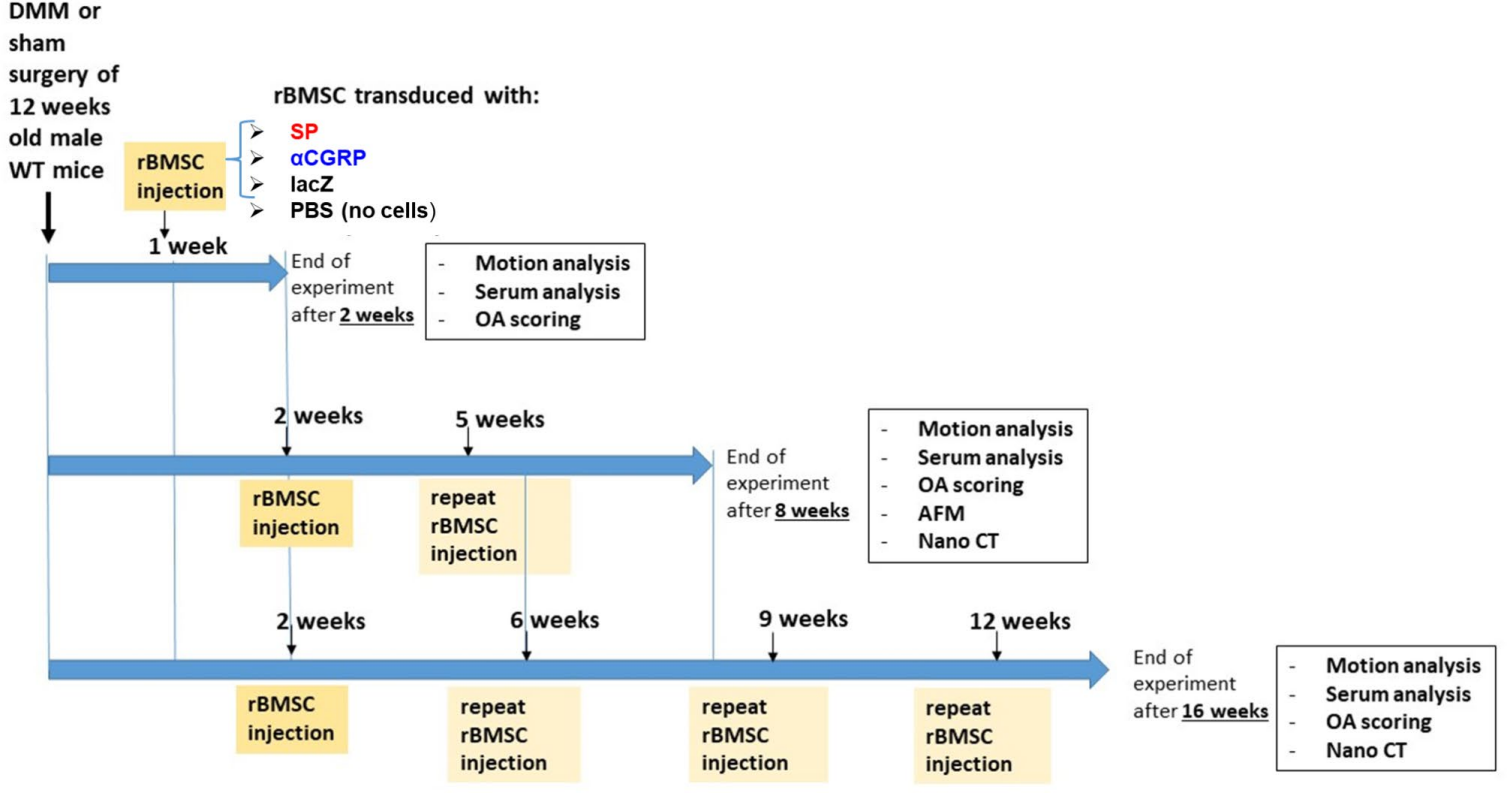
Scheme of animal experimental time lines. 12 weeks old male C57Bl/6J (WT) mice were subjected either to DMM or sham surgery and randomly divided into four groups (SP, αCGRP, lacZ or PBS) each according to type of injection post-surgery. At indicated time points, these groups were subjected to intra-articular (i.a.) injection of 350.000 of either rBMSC-SP, rBMSC-αCGRP, rBMSC-lacZ (in 7 µl PBS) or 7 µl PBS (no cells). At longer follow up times, i.a. injections were either repeated 1 × (8 weeks: total of 2 injections) or 3 × (16 weeks: total of 4 injections). Follow up was finished either after two weeks (early OA stage), eight weeks (full OA stage) and 16 weeks (late OA stage). A day before euthanasia, mice were subjected to motion analysis and directly before euthanasia blood removal for serum marker analysis. After euthanasia, knees were removed for OA scoring, Atomic Force Microscopy (AFM) analysis and nano-CT analysis

1 or 2 weeks after DMM or sham surgery (see experimental overview, Fig. 1), mice were subjected to intraperitoneal anesthesia with ketamine and xylazin (120 mg/kg b. wt. and 8 mg/kg b. wt). Then, 7 µl PBS without cells or 350.000 rBMSC (expressing either SP or CGRP or lacZ) resuspended in 7 µl PBS were injected i.a. into the surgically treated right knee (DMM or sham). Injection was repeated in the 8 and 16-weeks groups as shown in overview Fig. 1.

At the end of the experimental time line which is after 2, 8, and 16 weeks, animals were anesthesized (see anesthesia regimen above) and blood was collected via retro-orbital puncture and stored for serum analysis. Immediately after blood removal when still under deep anesthesia, the animals were killed by cervical dislocation. Knee joints were prepared and stored at -80 °C for nanoCT, AFM and histological analysis.

### Isolation and culture of BMSC

Bone marrow derived mesenchymal stem cells (rBMSC) were isolated from the bone marrow of femurs and tibias of 6 week old CD rats killed by using CO_2_ (Charles River, Sulzfeld, Germany). The cells were cultured in monolayer until reaching ∼80% confluency according to the method established previously from our group [16].

### Plasmid generation for lentiviral transduction

A lentiviral transduction system (ViraPower™ Lentiviral Expression System, Invitrogen, Carlsbad, CA, USA) was established for the generation of stable expression of murine SP, murine αCGRP or lacZ in rBMSC.

Therefore, we cloned the full-length SP (NM_009311.2) or αCGRP (NM_007587.2) *murine* cDNA sequence 3′ to a CMV promoter, into a lentiviral expression vector (pLenti6/V5-D-TOPO, Invitrogen, Carlsbad, CA, USA). As a neuropeptide control, we used the lentiviral vector backbone expressing the bacterial lacZ cDNA (pLenti6/V5-DEST Gateway Vector, # V49610, Invitrogen, Carlsbad, CA, USA).

### Lentivirus preparation and transduction

We co-transfected a 293FT producer cell line with the SP-, αCGRP- or lacZ-pLenti expression plasmids together with the ViraPower Packaging Mix. After 48 h, the viral supernatant was harvested and the virus titer was determined. Lentiviral particle were stored at -80 °C until transduction of rBMSC.

For the transduction, rBMSC were cultured until passage 5 in Minimum Essential Medium Eagle alpha (#M4526, Sigma-Aldrich Chemie GmbH, Taufkirchen, Germany) with 10% FBS (#S0615, Sigma-Aldrich Chemie GmbH, Taufkirchen, Germany), 1% antibiotics/antimycotics (#A6964, Sigma-Aldrich Chemie GmbH, Taufkirchen, Germany) and 2mM L-Glutamine (#35050-037, Thermo Fisher Scientific, Waltham, MA, USA). The viral supernatant was divided 1:2 and 1:5 and added to 50.000 rBMSC per well cultured in a 6-well plate. After 16–24 h, the lentiviral particles were removed and the rBMSC were further cultured. Selection was performed via addition of 2 µg/ml Blasticidin (InvivoGen, Toulouse, France) into the culture medium for 28 days. To verify successful transduction and subsequent (over-) expression of SP and αCGRP, ELISA and qRT-PCR was conducted with a small aliquot of the transduced rBMSC (qRT-PCR and ELISA) and the appropriate culture medium supernatant (ELISA) and compared to the rBMSC-lacZ group. The remaining cells were frozen in liquid nitrogen together with 10% DMSO (Carl Roth, Karlsruhe, Germany) until i.a. injection.

### ELISA

Blood samples from mice were centrifuged and serum was collected and stored at -80 °C until analysis. Enzyme-linked immunosorbent assays (ELISA) were used to measure expression and secretion of the neuropeptides SP (#ADI − 900 − 018, Enzo Life Sciences, Farmingdale, NJ, USA) and αCGRP (#EK − 015 − 09, CGRP rat/mouse Enzyme Immunoassay (EIA) Kit, Phoenix Pharmaceutical Inc., Burlinghame, CA, USA) in the cell lysate and supernatant of transduced rBMSC, according to the manufacturer’s instructions. Adiponectin ELISA (#ELM-Adiponectin-1, Mouse Adiponectin ELISA Kit, Ray-Biotech, Peachtree Corners, Georgia, USA) was used to measure the adiponectin level in the serum of DMM and sham mice.

### Luminex analysis

Using the individual designed ProcartaPlex Mouse and Rat Mix and Match (Thermo Fisher Scientific, Waltham, MA, USA) the following targets were analysed in the serum of the mice: IL-1 beta, IL-10, IL-17 A, IL-4, IL-6, Leptin, MCP-1, MIP-1 alpha, MIP-1 beta, RANKL, TNF alpha and VEGF-A according to manufactures protocol.

The measurement was carried out with the MAGPIX (Luminex Corperate, Austin, TX, USA) of the Institute of Medical Microbiology and Hygiene, University Hospital Regensburg.

### RNA isolation and real-time -PCR

Total cellular RNA was isolated from rBMSC using the Absolutely RNA Miniprep Kit (Stratagene, San Diego, CA, USA) according to the manufacturer’s instructions. To generate single-stranded cDNA, RNA was reverse transcribed with an AffinityScript QPCR cDNA Synthesis Kit (Stratagene, San Diego, CA, USA) and PCR was performed with the Mx3005P QPCR System from Agilent Technologies using Brilliant II SYBER Green qPCR Mastermix (Agilent Technologies, Santa Clara, CA, USA). Gene expression of transduced (SP or αCGRP) rBMSC was analyzed relatively, calibrated to the expression in control cells (lacZ), and normalized to 18s RNA using following primer (Table 1)

**Table 1:** qPCR primers

Gene	Forward	Reverse
SP	TTGGATTAATGGGCAAGCGG	TTCGTAGTTCTGCATCGCG
αCGRP	CAGATCTAAGCGGTGTGGGA	CCTTGGCCACATCCCTTTTC
*18s*	AAACGGCTACCACATCCAAG	CCTCCAATGGATCCTCGTTA

### Histology and OA-scoring

Knee samples were fixed in 4% paraformaldehyde/PBS for 16 h and decalcified in 20% EDTA/PBS, pH 7,4 for 5 weeks. Samples were embedded in paraffin and 6 μm frontal sections of the knee joint were taken using a microtome (RM255, Leica, Wetzlar, Germany). For evaluation of cartilage degradation, 5–6 sections in 70–120 μm intervals were deparaffinized, rehydrated and stained with Safranin O, Weigerts iron haematoxylin (Carl Roth, Karlsruhe, Germany) and Fast Green (Applichem, Darmstadt, Germany). Scoring was performed by two blinded, independent observers according to the OARSI guidelines [17] with little modifications (Table 2). Sections were scanned with 10x magnification using the TissueFAXS system from TissueGnostics (DFG code: INST 89/341-1 FUGG; Vienna, Austria). Maximum OARSI scores of the medial/lateral femur condyles and the medial/lateral tibia plateau were averaged and are displayed in the graphs.

**Table 2:** OARSI guidelines for assessment of murine cartilage degradation

Grade	Osteoarthritic damage
0	Normal
0,5	Loss of Safranin O staining without structural changes
1	Small fibrillations without loss of cartilage / uneven surface
2	Vertical clefts and erosions down to the layer immediately below the superficial layer and some loss of surface lamina
3	Vertical clefts / erosions to the calcified cartilage < 25% of the articular surface
4	Vertical clefts / erosions to the calcified cartilage 25–50% of the articular surface
5	Vertical clefts / erosions to the calcified cartilage 50–75% of the articular surface
6	Vertical clefts / erosions to the calcified cartilage > 75% of the articular surface

### PCR-based detection of intra-articular injected rBMSC in mouse knees

To detect i.a. injected rBMSC in mouse knee joints, we isolated total DNA from paraffin sections of mouse knees with i.a. injected rBMSCs using the High Pure PCR Template Preparation Kit (Roche, Basel, Switzerland) according to manufacture manual. Specific primer for different vomeronasal receptors, which can only be found in mouse or rat were used to identify the rat DNA in the isolated DNA of the mouse knee sections. 100ng of DNA were used for performing end-point-PCR and the bands were visualized on a 2% agarose gel (Table 3).

**Table 3:** Endpoint PCR primers

Gene	Forward	Reverse
V1rm1 rat	TGGCTTTCAGGCCACCAGGC	GCTCTGTCCTCAGGGGCAGGT
V1rm 2 rat	AGAAGAGTACTGCCCAAGGGACA	GGGGCTGAACGCTGGGAAGC
V1rh3 mouse	GGGAGGGGCAGTGCTACAT	TGCCACCAATCAACCAGAAGCCCA
V1rh10 mouse	TTCAGGGTGCTATGGGAGGGGC	GCCCATCCCTGTGAATCAGCACA

### Motion analysis

2, 8 and 16 weeks after DMM or sham surgery, mice were subjected to video-tracking motion analysis to determine alteration in mobility. Up to 9 mice at a time were observed and filmed for 1 h. The second half of the film (last 30 min) was analyzed for the distance the mice moved (in cm) and the velocity (cm/s) using Ethovision XT software (Noldus, Wageningen, Netherlands).

### Indentation–type atomic force microscopy

Atomic force microscopy was solely used in the indentation mode. For indentation-type atomic force microscopy (IT-AFM), native, non-fixated knee tissues 8 weeks after DMM- or Sham-surgery were embedded in OCT tissue freezing medium, snap frozen in isopentane, chilled in a liquid nitrogen bath and cut in frontal direction into 20 μm slides at -20 °C using a cryotome (Leica CM 1950, Leica Biosystems, Nussloch, Germany). To maintain tissue integrity throughout AFM measurements, transparent adhesive tape (tesafilm Nr.: 57330-00000, Tesa, Norderstedt, Germany) was used to obtain the tissue sections which were then attached to a glass slide via a double adhesive tape (tesafilm Nr.: 56661-00002).

IT-AFM was carried out using a NanoWizard I AFM (JPK Instruments, Berlin, Germany) in combination with an inverted optical microscope (Axiovert 200, Carl Zeiss Micro Imaging GmbH, Göttingen, Germany) for positioning of the AFM-tip on sample surface. The influence of external noise was reduced by placing the whole set-up on an active vibration isolation table (Micro 60, Halcyonics, Göttingen, Germany) inside a custom made 1 m³ soundproof box. Indentation experiments were performed with silicone-nitride cantilevers (MLCT, Cantilever E, Bruker, Paris, France) with a nominal spring constant of 0.1 N/m, a nominal tip radius of 20 nm and a pyramidal tip shape. For each cantilever the spring constant was determined individually using the thermal noise method [18]. During measurements, the tissue sections were immersed in PBS (Bio&Sell (D) PBS, without Ca2 + and Mg2+, Feucht, Germany).

The frontal cut of the knee tissues allowed a clear identification of the medial tibia plateau and its individual cartilage zones (superficial, middle and deep zone) using the 20x Objective (LD A-Plan, 20x/NA 0.3 Ph1) of the inverted optical microscope of the AFM setup. All three zones were investigated by IT-AFM using the frontally cut sections right after defrosting them at room temperature. Each recorded force map contained 25 × 25 force-indentation curves equally distributed over an area of 3 × 3 μm². The vertical tip velocity was 15 μm/s throughout all IT-AFM measurements. In total, 6 × 625 force-curves for each cartilage zone, each surgery type (DMM, sham) and each injection type (PBS, lacZ, SP, αCGRP) were assessed by using two different sections per animal. For the following analysis of the recorded force-curves, the CANTER Processing Toolbox (https://github.com/CANTERhm/CANTER_Processing_Tool) was used. After converting the force vs. z-piezo position curve to a force vs. indentation curve, the Young’s Modulus was extracted by fitting the Hertz-Sneddon model for a pyramidal indenter [19, 20] to the approach part of the force-indentation curves up to an indentation depth of 1 μm (a detailed description of these steps can be found in Hartmann et al. [21]. Then, Young’s modulus (stiffness) distributions were generated, and the two maxima of the bimodal distributions were determined by fitting a linear combination of two Gaussian distributions to the data [22]. Loparic et al. observed a similar bimodal nano-stiffness in mature articular cartilage and demonstrated that the first peak can be attributed to the proteoglycan phase and the second peak to the collagen fibrils [23].

### Nano-CT

The nano-CT analysis procedures conducted in this study closely mirror those employed in our previous investigations [13, 24, 25]. Knee joints were collected at two different time points, 8 weeks and 16 weeks post-surgery, and initially preserved in 4% paraformaldehyde (PFA) for 16 h. After fixation, the joints were stored in a 70% ethanol solution at 4 °C.

For imaging, knee joints were subjected to scanning using a Scanco µCT 50 device (Scanco Medical, Brüttisellen, Switzerland) while submerged in 70% ethanol. The imaging settings included a source voltage of 90 kVp and an intensity of 88 µA. To minimize beam hardening effects, a 0.50-mm-thick aluminum filter was employed. Two sets of scans were conducted: one with an isotropic voxel size of 6.8 μm and 800 ms integration time, intended for acquiring 3D overviews of the femorotibial joint, assessing topographical changes in the subarticular region, meniscal ossicles, and potential osteophyte formations. A higher resolution scan, with a 2.0 μm voxel size and 1500 ms integration time, was performed in a narrower region of each sample to capture sharper details. Reconstruction of the acquired images was carried out using Scanco’s OpenVMS software.

Bone morphometry indices were calculated within two volumes of interest (VOIs): the first VOI encompassed the subarticular region, specifically a 1.2 mm³ region located 300 mm distal from the epiphyseal line for evaluating sub-articular trabecular morphometry. The second VOI covered an approximate 0.2 mm³ area within the medial epiphysis, situated between the lower margin of the subchondral bone plate and the epiphyseal line. In both cases, optimal threshold settings, in accordance with Scanco’s OpenVMS software (lower threshold: 685.3 mg HA/cm³, upper threshold: 3000 mg HA/cm³, Gauss Sigma: 0.8, Gauss Support: 1), were implemented. Manual contouring was carried out while excluding the endocortical surface, adhering to standard guidelines.

The segmentation of calcified cartilage (CC) from the underlying subchondral bone plate was performed through a semi-automatic process, which involved generating two initial point clouds using lower and upper threshold values set at 396.0 and 933.0 mg hydroxyapatite (HA)/cm³, respectively. This process was further refined with a modified Seeded Region Growing technique [26]. Colormaps representing CC thickness were subsequently generated for the medial condyle, with scaling applied to achieve a maximum value of 80 μm for all samples.

In addition, the thickness of the subchondral bone plate was quantified using ImageJ software in three evenly spaced coronal cross-sections of the joint. The intercondylar eminence was excluded from this measurement, and the final values reported for each condyle were expressed as the mean ± SEM of 60 spots across the three coronal planes.

The assessment of tibial plateau morphology involved measuring the length of the lateral and medial condyles, defined as the distance from the center of the condyle near the trochlear groove to the lateral or medial prominence.

To investigate heterotopic ossification of the meniscus, anterior meniscal ossicles were manually delineated and segmented. The volumes of interest (VOIs) for Sham and DMM mice were approximately 0.4 mm³ and 1.0 mm³, respectively, accounting for the irregular surface expansion of the ossicles due to post-traumatic changes. Quantitative analysis included bone volume (BV), bone mineral density (BMD), bone surface (BS), and the BS/BV ratio for the anterior meniscus, and comparisons were made across the different target groups.

### Statistics

Statistical analysis was performed using Prism 6 (GraphPad Software Inc., San Diego, CA, USA). Results are presented as the means ± standard deviation (SD). Each assay was performed in biological and technical replicates and repeated at least in 3 independent experiments. Two-tailed Mann–Whitney tests were used as standard nonparametric tests, determining whether the medians of the two groups (experimental group vs. control) were significantly different. Exact p-values were calculated. A value of *p* ≤ 0.05 was considered statistically significant.

For the data derived from nanoCT, a Generalized Linear Model (GLM) was used for statistical analysis, taking into account a factorial design with three independent variables: age group (8 weeks or 16 weeks), surgery type (sham or DMM), and treatment substances (PBS, lacZ, SP, αCGRP). The GLM was composed of a linear regression model, which was designed to analyze the impact of the three main predictors (age, surgery type, treatment substance), as well as their interactions on the measured outcome variables. In case a significant effect for independent variables was observed, pairwise comparison was conducted between each level of the independent variables with the Bonferroni adjustment. The significance level was set at α = 0.05.

## Results

### Generation of lacZ-, SP- and αCGRP-(over)expressing rBMSC and isolation of DNA for detection of intra-articular injected cells

**Fig. 2:**
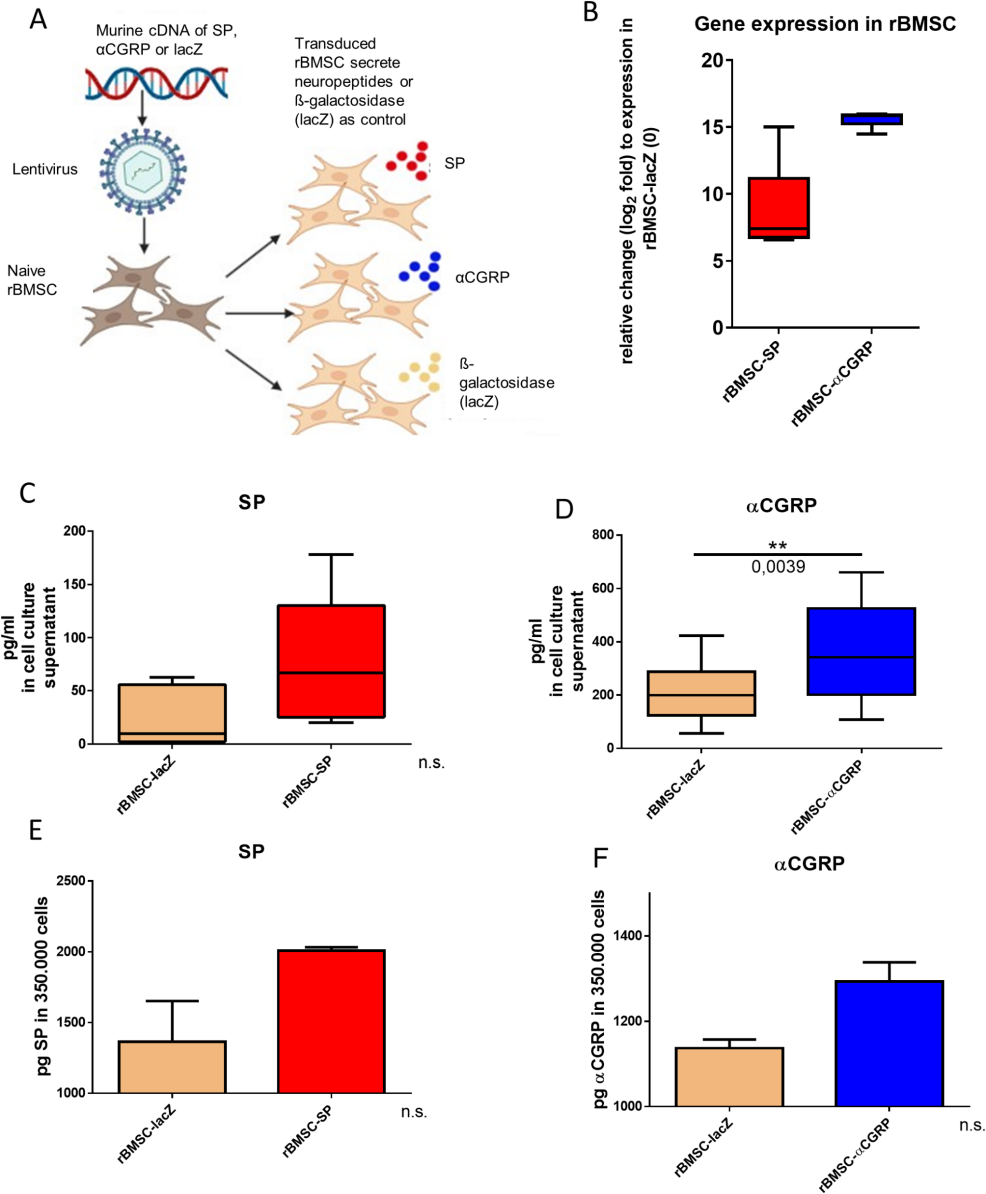
Transduction of rBMSC and gene and protein expression of SP and αCGRP. **A**) Murine cDNA for SP, αCGRP and lacZ was cloned into lentiviral expression vectors, and viral particle were generated using a 293FT producer cell line. Subsequently, naive rBMSC were transduced with these viruses to produce lacZ-, αCGRP- or SP (over-) expressing rBMSCs clones. **B**) Gene expression analysis for SP and αCGRP was performed to verify expression level in transduced rBMSC in comparison to lacZ control rBMSC. **C**, **D**) SP and αCGRP protein concentration was determined in the cell culture supernatant of transduced rBMSC and adjusted to the secreted level from rBMSC-lacZ. **E**, **F**) SP and αCGRP protein concentration was determined in 3.5 × 10^5^ transduced rBMSC, which corresponds to the number of cells per i.a. injection. Protein concentration of the neuropeptides was compared to that of the rBMSC-lacZ group. Results are means +/- SD; one sample t-test **p ≤ 0,01; *N* = 7–9

We transduced rBMSC with the cDNA of the reporter gene lacZ (rBMSC-lacZ) or murine αCGRP cDNA or murine SP cDNA using a Lentiviral system (ViraPower), and performed selection with Blasticidin for 14 days to generate rBMSCs stably expressing lacZ, αCGRP or SP (Fig. 2A). Gene expression analysis revealed a more than 500 fold increase (log2 fold change = 9) of SP mRNA expression and a more than 35.000 fold increase (log2 fold change = 15) increase of αCGRP gene expression in comparison to the lacZ control cells (Fig. 2B). ELISA analysis of the cell culture supernatant showed that SP-primed rBMSC (rBMSC-SP) produced and released more SP as the rBMSC-lacZ controls by trend, whereas αCGRP primed rBMSC (rBMSC-αCGRP) released significantly more αCGRP compared to the lacZ control cells (2 times more) (Fig. 2C, D). Analysis of cell lysates revealed that both SP- and αCGRP-rBMSC clones produced more neuropeptides compared to the rBMSC-lacZ group. Protein concentration was adjusted to 3.5 × 10^5^ cells, which corresponds to the number of injected cells per knee (Fig. 2E, F).

To track the location of the i.a. injected rBMSCs, we isolated the total DNA from the knee paraffin-embedded sections and amplified a sequence in the gene for vomeronasal receptors using mouse and rat specific primer. Supplementary Fig. 1 shows a representative agarose gel image (suppl. Figure 1). The mouse primers generated bands when using DNA from knee sections of the 4 treatment groups (B, lanes 1–4), however when using the rat primers, no band was generated indicating absence of rat DNA (A, lanes 1–4). When using DNA extracted from the 3 injected rBMSC groups for PCR as rat primer control, PCR products were generated (A, lanes 5–7) whereas mouse primers did not generate a product (B, lanes 5–7).

### Scoring of articular cartilage matrix changes after DMM/sham surgery and i.a. rBMSC injection

**Fig. 3:**
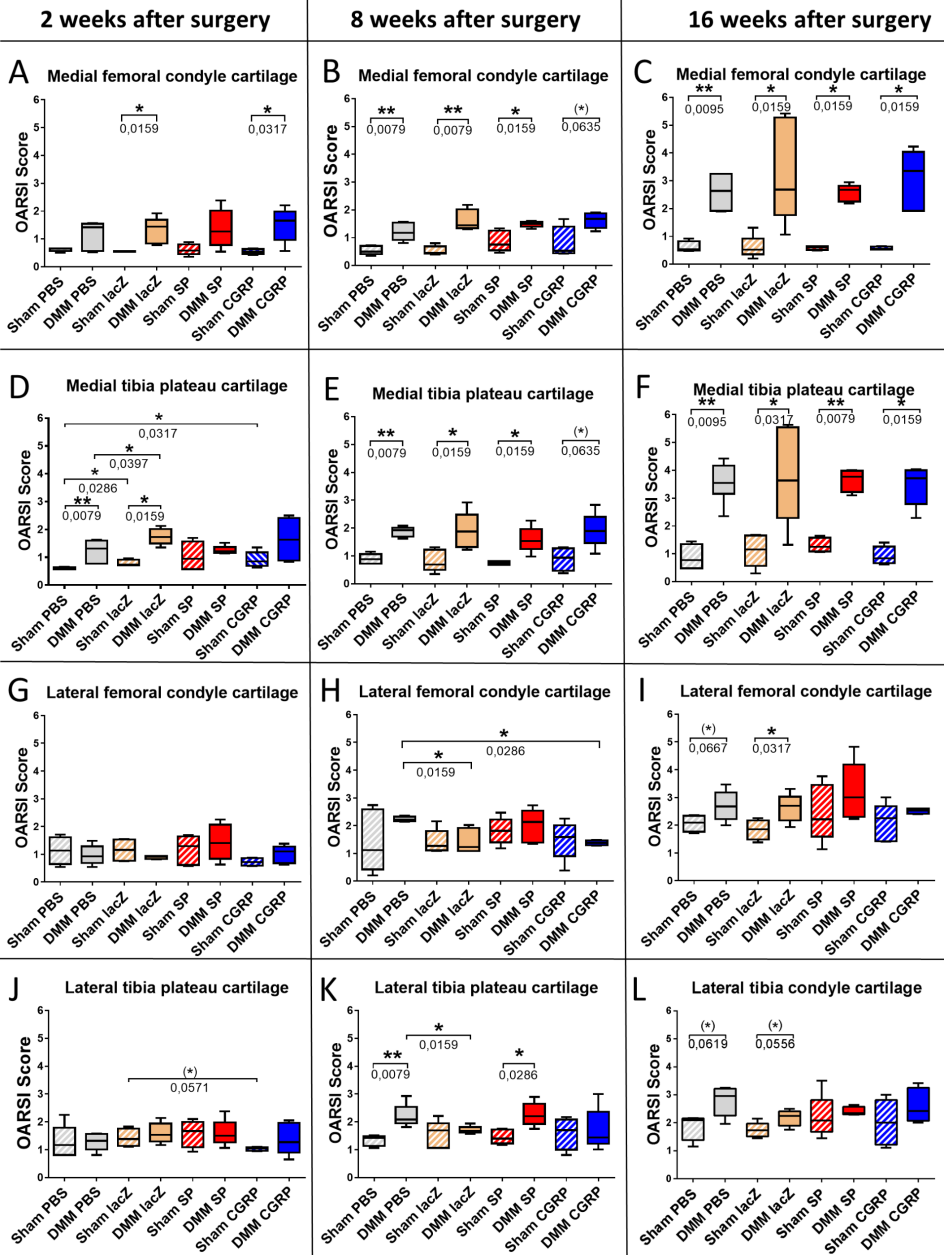
OARSI scores of rBMSC treated mice 2, 8 and 16 weeks post DMM or sham surgery. Comparison of the means of the OARSI scores of the articular cartilages from the (**A**-**C**) medial femoral condyle, (**D**-**F**) medial tibia plateau, (**G**-**I**) lateral femoral condyle and (**J**-**L**) lateral tibia plateau from all four treatment groups, 2, 8 and 16 weeks after either DMM or sham surgery. Sham/DMM lacZ = rBMSC-lacZ group; Sham/DMM SP = rBMSC-SP group; Sham/DMM αCGRP = rBMSC-αCGRP group; 2-way ANOVA; *p ≤ 0,05; **p ≤ 0,01; *N* = 4–6

Cartilage matrix structure was evaluated in safranin O stained knee joint sections throughout all groups and exp. time points. Figure 3 presents an overview of the OARSI scores of the medial femoral condyles, medial tibia plateaus, lateral femoral condyles and lateral tibia plateaus from mice, 2, 8 and 16 weeks after either DMM or sham surgery and subsequent i.a. injections of primed rBMSCs. OARSI scores were significantly increased in almost all DMM groups in comparison to the respective sham animals in the medial femoral condyles and medial tibia plateaus except for some groups at the early 2 weeks time point (Fig. 3A– F), indicating successful OA induction via DMM surgery. However, no differences between the four treatment groups were detected in the medial cartilage knee regions, except at 2 weeks past surgery (Fig. 3D). Here, the difference between both sham and DMM surgery was statistically significant in the control groups (PBS and rBMSC-lacZ), and between the αCGRP- and PBS sham groups. Interestingly, 2 weeks after surgery, there was no significant difference in OARSI score concerning cartilage destruction of the medial femoral condyles and tibia plateau in the SP-group and the medial tibia plateau of αCGRP group between DMM and sham animals (Fig. 3A, D).

For the 2 week lateral groups, no differences between DMM and Sham surgery were detectable in the lateral femoral and tibial cartilage (Fig. 3G, J). However, the tibia score was decreased by trend in the αCGRP sham treatment group compared to the lacZ sham group (Fig. 3J). The OARSI score of lateral femoral condyle cartilage was significantly decreased in 8-weeks-DMM animals treated with rBMSC-αCGRP, compared to DMM-PBS control animals (Fig. 3H) and between the DMM rBMSC-lacZ and PBS groups (Fig. 3H, K). At the 16 weeks time point, except for the rBMSC-SP and the rBMSC-αCGRP groups, the lateral femoral condyle and lateral tibia plateau cartilage of the other groups showed alteration of OARSI scores between DMM and sham surgery groups by trend. At this late time point, no score differences were observed between the four treatment groups anymore (Fig. 3I, L).

Representative safranin O stainings, demonstrating the severity and location of the cartilage damage in the mice knee joints for all time points and all locations, are shown in suppl. Figure 2 A, B.

### Cartilage molecular biomechanical properties analysed by AFM

**Fig. 4:**
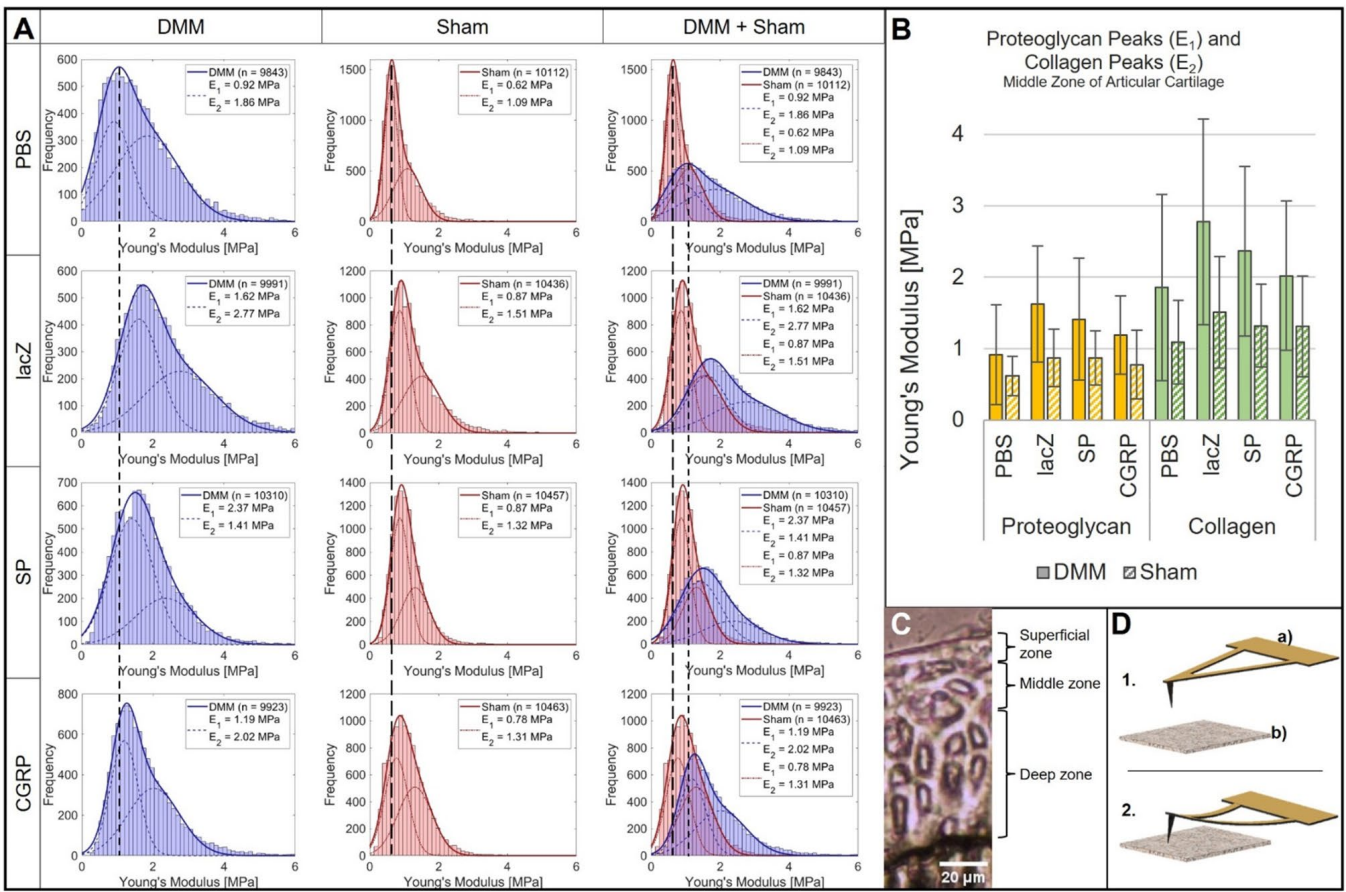
IT-AFM-based analysis of articular cartilage matrix stiffness of the middle cartilage zone (MZ) at 8 weeks post DMM or sham surgery. **A**) Histograms of Young’s modulus (stiffness) distributions of the MZ cartilage matrix of mice 8 weeks after DMM or sham surgery and injection of either PBS, lacZ-, αCGRP- or SP-expressing rBMSC. The continuous red or blue lines in each histogram represent a fit to the data using a linear combination of two Gaussian distributions; the dashed red or blue lines show the individual Gaussian distributions representing the proteoglycan (left) and the collagen (right) Young’s moduli, respectively. Dashed black lines between all histograms within one surgery type (sham or DMM) indicate peak shifts due to each rBMSC-injection compared to the control PBS injection. *N* = 3. **B**) Mean Young’s modulus (stiffness) of the proteoglycan peak and the collagen peak of the MZ cartilage at 8 weeks after DMM or sham surgery and i.a. injection of either PBS, lacZ-, αCGRP- or SP-(over)expressing rBMSC. Bars show mean ± standard error of the mean. 2-Sided t-test of independent samples between types of surgery (per injection within the MZ) and injection vs. injection (per type of surgery within the MZ). *N* = 3. **C**) Overview phase contrast optical microscopy image of a frontal cryosection of native (non-decalcified) mouse articular cartilage indicating the different cartilage zones (SZ, MZ, DZ) included into the analysis. **D**) Scheme shows the principle of IT-AFM on articular cartilage in two steps: (1) The cantilever (a) holding the tip on its underside approaches the cartilage sample (b). (2) As soon as contact between tip and sample is established the cantilever bends while the tip indents the sample. The deformation of the cantilever is detected via a laser pointed at its upper side and recorded together with the vertical displacement for further analysis

A biomechanical analysis of the extracellular matrix (ECM) of the medial tibia plateau in mice 8 weeks after surgery is shown in Fig. 4A-D. Here, the stiffness distributions of the middle zone (MZ) were displayed representatively for the other two zones, the deep zone (DZ) and the superficial zone (SZ). The four different treatment and two different surgery groups revealed differences in matrix stiffness. Within each treatment group in the MZ, the stiffness of the DMM group was consistently higher compared to the respective sham group indicated by the shift to the right of the DMM stiffness distribution compared to the respective sham distribution. Comparing the four treatment groups within the DMM surgery, the mice treated with rBMSC-lacZ seemed to have the highest cartilage stiffness in the MZ followed by the rBMSC-αCGRP/SP groups and the lowest stiffness was observed in the PBS group. When comparing the sham groups, the PBS treated group revealed again the lowest cartilage stiffness. The treatment with rBMSC-SP/αCGRP showed no significant differences between the two neuropeptides, and the rBMSC-lacZ group appeared to have the largest shift to the right and therefore the highest stiffness in the sham group. However, the differences between the three rBMSC treatment groups in the sham surgery group were significantly less compared to the differences in the DMM groups.

In the DZ (Suppl. Figure 3 A, B), stiffening of the DMM group compared to the sham group was observed in the rBMSC-lacZ and rBMSC-αCGRP groups, the rBMSC-SP groups and seemed to be not different between DMM and sham. The PBS treated DMM group appeared to be softer than the sham group. However, the DMM PBS group did not show the typical positively skewed bimodal distribution. Within the DMM group, the DZ of mice treated with rBMSC-SP and rBMSC-αCGRP displayed a lower stiffness compared to the PBS group. The highest stiffness was observed in the rBMSC-lacZ group, which had the largest shift of the curve distribution to the right. Regarding the sham animals, all three rBMSC groups appeared to be softer compared to the PBS group, with the rBMSC-αCGRP group being the softest one.

In the SZ (Suppl. Figure 4 A, B), the stiffening of the DMM group compared to sham was detected in the three rBMSC treatment groups. All DMM groups displayed an atypical bimodal distribution, in particular the PBS group, which showed a distinct narrow peak at low stiffness. In the sham group, the rBMSC-αCGRP group was slightly softer than the rBMSC-SP group, but both treatment groups revealed a lower stiffness compared to the PBS and rBMSC-lacZ groups.

### Motion analysis

**Fig. 5:**
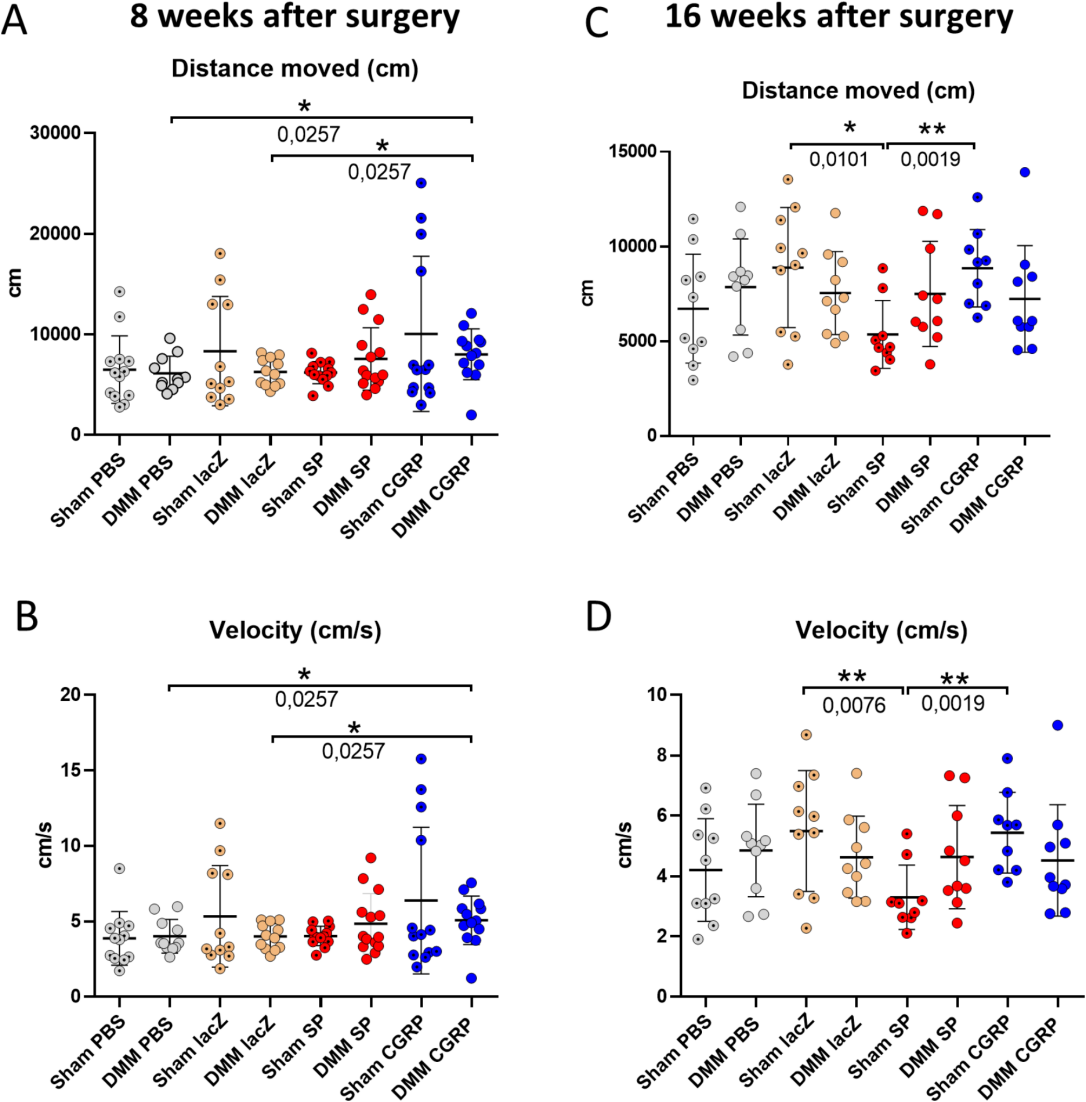
Motion analysis of rBMSC-treated mice 8 and 16 weeks post DMM or sham surgery. 8 and 16 weeks after DMM or sham surgery, mice were subjected to video-tracking motion analysis to determine mobility activity. The mice were analyzed for the moved distance (in cm) (**A**, **C**) and the velocity (cm/s) (**B**, **D**) using Ethovision XT software. Sham/DMM lacZ = rBMSC-lacZ group; Sham/DMM SP = rBMSC-SP group; Sham/DMM αCGRP = rBMSC-αCGRP group; Mann-Whitney-test; *N* = 11–13; *p ≤ 0.05; **p ≤ 0.01

We analyzed the movement and activity of the four treatment groups, 8 and 16 weeks after DMM or sham surgery. Therefore, mice were video tracked for 60 min, and subsequently EthoVision XT software was applied for video tracking and analysis of the mobile behavior for the last 30 min of the filming. The evaluation of the video tracking data revealed, that 8 weeks post OA-induction, DMM-mice treated with rBMSC-αCGRP showed a significant increase in the distance they moved (Fig. 5A), as well as in the average velocity (Fig. 5B), in comparison to DMM mice treated with rBMSC-lacZ and with PBS. 16 weeks post-surgery, no significant difference was observed anymore between the DMM mice treated with the different rBMSC groups. However, the mobility of the sham animals in the three rBMSC treatment groups was altered. The moved distance (Fig. 5C) and the average velocity (Fig. 5D) of the sham-rBMSC-SP animals was significantly decreased compared to the sham-rBMSC-αCGRP and to the sham-rBMSC-lacZ animals.

### Subchondral bone properties analysed by Nano-CT

#### Medial subchondral bone plate thickness

**Fig. 6:**
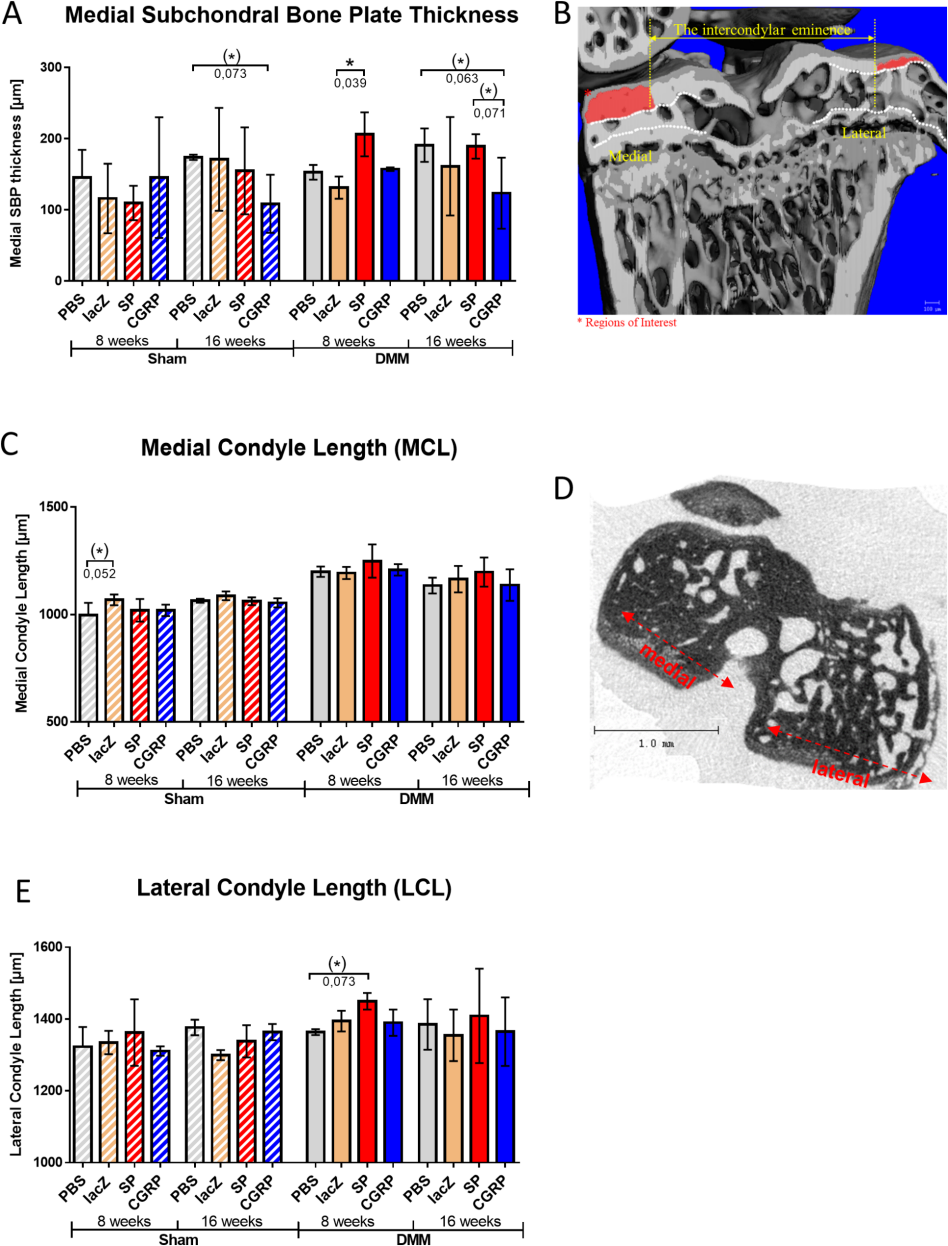
Nano CT analysis of medial subchondral bone plate thickness (SCBP) and the medial and lateral condyle length. (**A**) Quantification of the SCBP thickness in the control (PBS and rBMSC-lacZ) and the rBMSC-αCGRP- and–SP groups, 8 or 16 weeks after the DMM or sham surgery. Image on the right shows the representative image of the tibial plateau with the corresponding ROI marked by * and red bars. (**B**) Diagram of the medial condyle length and **C**) of the lateral condyle length in the different treatment groups, 8 or 16 weeks after the DMM or sham surgery. Representative µCT images display the tibial plateau, as well as an representatively measured condyle length. Generalized Linear Model (GLM); n (total) = 48; N (per group) = 3; *p ≤ 0.05; **p ≤ 0.01

NanoCT analysis illustrated, that the rBMSC-SP treated mice have a significantly increased medial subchondral bone plate (SBP) thickness at 8 weeks post DMM induction compared with DMM-lacZ control mice, and, by trend, also 16 weeks after DMM surgery compared with the rBMSC-αCGRP treated group. That could indicate either a more progressed OA stage in rBMSC-SP DMM mice or a more severe OA pathology. Notably, both the rBMSC-αCGRP treated sham and DMM groups revealed a tendency to a decreased SBP thickness at 16 weeks compared to the PBS treated control groups (Fig. 6A). The region of interest (ROI) for measuring the medial subchondral bone plate thickness is marked with red color in Fig. 6B.

The length of the medial condyles (MCL) was increased in all DMM groups compared to the sham groups. However, no differences between the DMM treatment groups were observed, only by trend between the lacZ and PBS sham groups at the 8 weeks time point (Fig. 6C). Lateral condyle length (LCL) was increased by trend in the rBMSC-SP treated groups at 8 weeks post DMM surgery compared to the PBS group (Fig. 6E). Figure 6D shows representative areas for determination of the condyle length.

The thickness of the medial calcified cartilage (CC) is generally increased in the DMM groups compared to their respective sham counterparts. Nevertheless, the effect of treatment was significant only for the rBMCS-SP sham group, which revealed an increased thickness of medial CC at 16 weeks after surgery compared with the PBS treated sham animals (suppl. Figure 5 A). A representative color map of the medial condyle is shown in suppl. Figure 5B.

All mice developed continuous osteophyte formations at the injury site (i.e. medial) both at 8 and 16 weeks, post-operative. Interestingly, already 8 weeks post DMM surgery, two out of three rBMSC-SP treated mice developed osteophytes on the lateral condyle as well. Likewise, in the rBMSC-αCGRP group, one mouse out of three developed osteophytes on the lateral side 16 weeks after the DMM surgery, while none of the mice from the other two DMM groups developed lateral osteophytes (suppl. Figure 6).

#### Medial epiphyseal morphometry

**Fig. 7:**
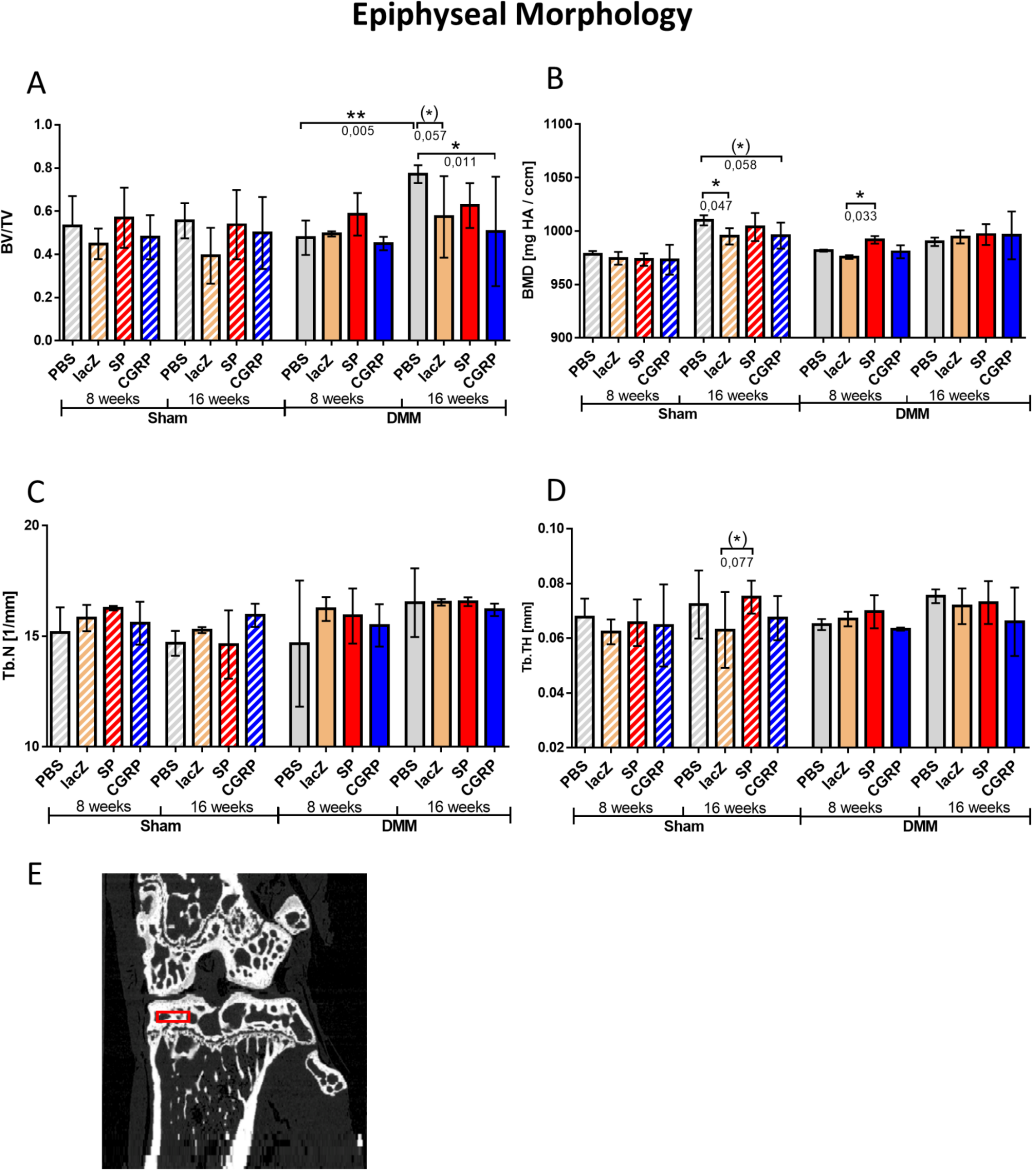
NanoCT analysis of the medial epiphyseal morphology. Diagram of the (**A**) bone volume fraction (BV/TV), (**B**) bone mineral density (BMD), (**C**) trabecular number (Tb.N.), and (**D**) trabecular thickness (Tb.Th.) in the epiphyseal bone region. A schematic illustration of the corresponding volume of interest (VOI) is depicted in (**E**). Generalized Linear Model (GLM); N (total) = 48; N (per group) = 3; *p ≤ 0.05; **p ≤ 0.01

The BV/TV increased significantly in the PBS treated DMM group between 8 and 16 weeks post-surgery. Conversely, there was no significant increase detectable in any of the rBMSC treated groups. Additionally, 16 weeks after the DMM surgery, the rBMSC-lacZ and -αCGRP groups had a significantly lower BV/TV compared to the PBS treated mice. Within the sham groups (both 8 and 16 weeks post surgery), no significant changes in BV/TV were observed within the four treatment groups (Fig. 7A). The BMD was reduced 16 weeks after sham surgery in the rBMSC-lacZ and–αCGRP groups compared to the PBS treated group. Within the 8 week DMM groups, a significant increase in BMD was observed in the rBMSC-SP treated group compared to the rBMSC-lacZ treated group (Fig. 7B). The trabecular number (Tb.N) and thickness (Tb.TH) of the medial epiphysis displayed neither significant alterations between the different treatment groups, nor between the DMM or sham surgery groups (Fig. 7C, D). Figure 7E indicates the volume of interest (VOI).

#### Subarticular bone morphometry

**Fig. 8:**
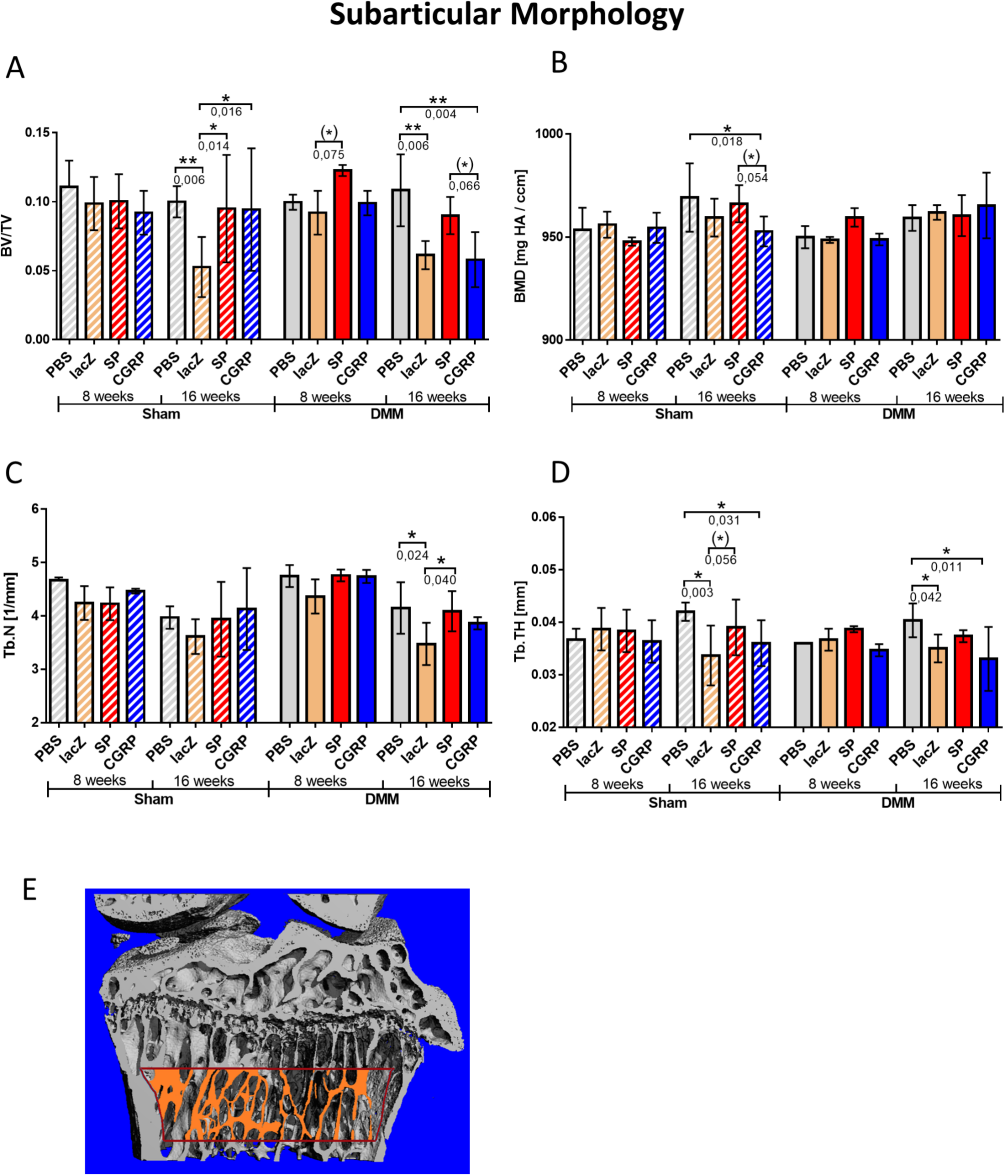
NanoCT analysis of the subarticular morphology. Diagram of the (**A**) bone volume fraction (BV/TV), (**B**) bone mineral density (BMD), (**C**) trabecular number (Tb.N.), and (**D**) trabecular thickness (Tb.Th.) in this region. A schematic illustration of the corresponding volume of interest (VOI) is depicted in (**E**). Generalized Linear Model (GLM); n (total) = 48; N (per group) = 3; *p ≤ 0.05; **p ≤ 0.01

Below the growth plate, in the subarticular region, treatment-specific changes in the bone volume fraction (BV/TV) were observed mostly 16 weeks post-operative. Within the sham groups, rBMSC-lacZ demonstrated the lowest BV/TV compared to all other treatment groups. In the 16 weeks DMM group, however, the highest bone volume fraction belonged to the rBMSC-PBS control group, with significant differences between the rBMSC-lacZ, and the rBMSC-αCGRP. The BV/TV of the rBMSC-αCGRP treated group was reduced by trend compared to the rBMSC-SP in the 16-week DMM groups. 8 weeks after the surgery, only the DMM rBMSC-SP treatment group had an increased BV/TV by trend compared to the rBMSC-lacZ treated group, and no other differences were observed between the treatment groups (Fig. 8A).

As for the effect of treatment on the BMD of the trabecular bone, a decrease was observed in 16 weeks sham groups between the rBMSC-αCGRP group and the PBS- and rBMSC-SP treated groups (Fig. 8B). Regarding subarticular trabecular parameters, the rBMSC-lacZ treated group had a lower Tb.N. 16 weeks after the DMM surgery compared to the PBS and rBMSC-SP groups (Fig. 8C). 16 weeks after sham and DMM surgery, the rBMSC-lacZ/αCGRP groups revealed a lower Tb.TH. compared to the PBS group. Notably, 16 weeks post sham surgery the rBMSC-αCGRP and the rBMSC-lacZ treatment groups had a lower Tb.TH compared to the PBS treated group (Fig. 8D). Figure 8E indicates the corresponding VOI.

The BV of the anterior meniscal ossicles increased in all treatment groups 16 weeks after DMM surgery compared to the 8 weeks time point. In the sham groups, only the rBMSC-αCGRP treated group developed an increased BV 16 weeks after surgery compared to the PBS and rBMSC-lacZ groups (suppl. Figure 7 A). Meniscal BMD remained unchanged in all four treatment groups at both time points and both surgeries (suppl. Figure 7B). BS was increased in all treatment groups 16 weeks after DMM surgery and in the sham rBMSC-αCGRP treatment compared to the rBMSC-lacZ and PBS groups (suppl. Figure 7 C). BS/BV was decreased in all rBMSC treatment groups compared to the PBS control group 8 weeks after sham surgery. All other groups were unaffected (suppl. Figure 7D).

#### Determination of inflammation markers in the serum after surgery

**Fig. 9:**
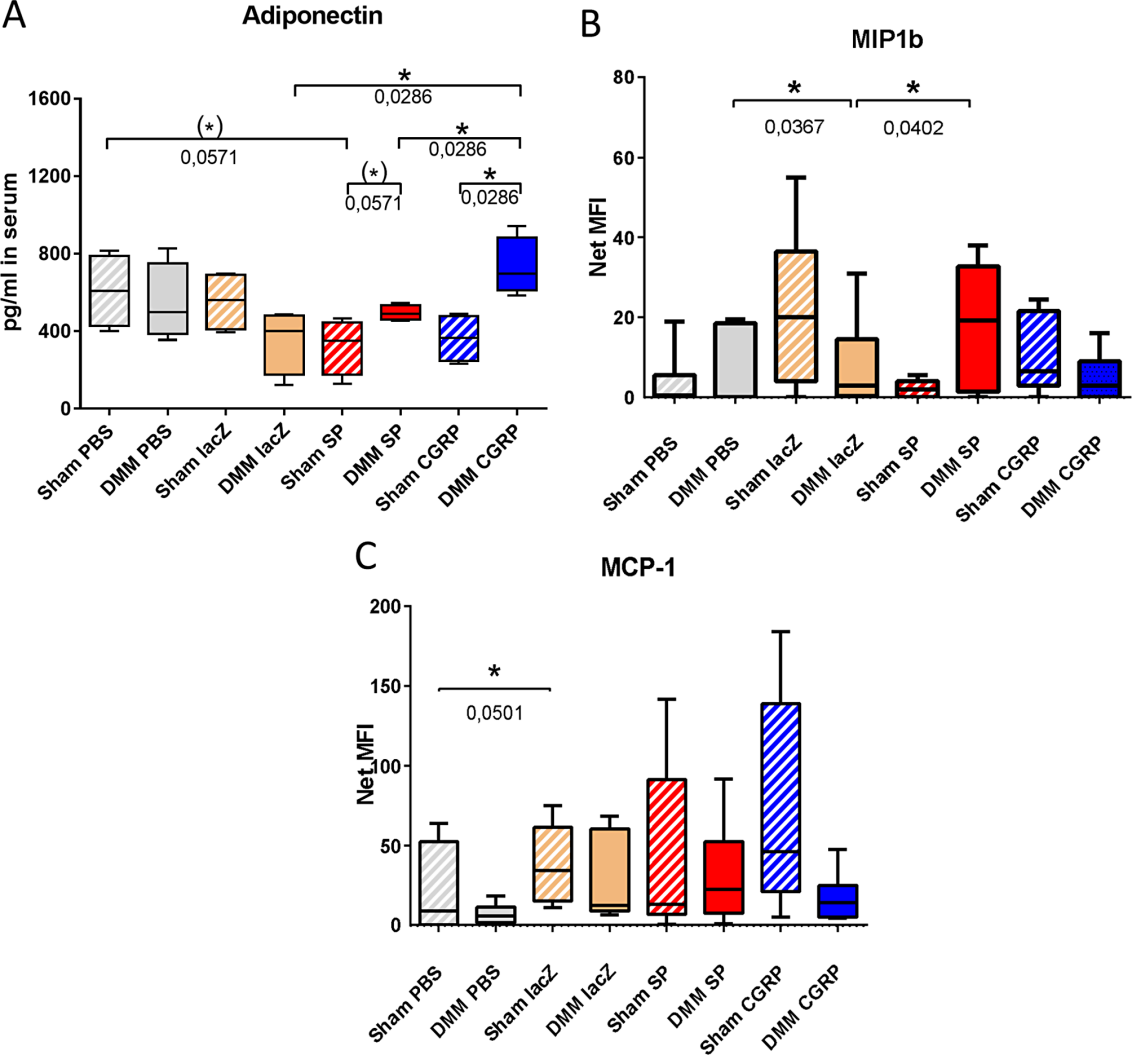
Serum concentrations of adiponectin, MIP1b and MCP-1 at 16 weeks after DMM/Sham surgery. Concentration of adiponectin (**A**), and concentration of MIP1b (**B**) and MCP-1 (**C**) in the serum of the four treatment groups 16 weeks after DMM or Sham surgery. Mann-Whitney p* ≤ 0,05; *N* = 3–4

We analyzed a panel of OA related inflammation and pain markers in the serum of DMM and sham mice. The analyzed cytokines were adiponectin, measured via ELISA, and Rankl, IL-1ß, IL-4, IL-6, IL-10, IL-17 A (CTLA-8), leptin, MIP-1a (CCL3), MIP-1b (CCL4), TNFα, VEGF-A, IFNγ, measured via Luminex multiplex analysis (Fig. 9). Only a few of these markers were regulated in the different treatment groups related to treatment and type of surgery. With respect to surgery, the adiponectin level was increased in the rBMSC-SP/αCGRP treatment groups 16 weeks after DMM surgery compared to the sham groups. With respect to the treatments, the adiponectin serum level of the DMM-rBMSC-αCGRP group was increased compared to the DMM-rBMSC-lacZ treatment group. Besides, the adiponectin serum level was higher in the sham-PBS group compared to the sham-rBMSC-SP treatment group (Fig. 9A). MIP1b level was increased after DMM in the rBMSC-SP group compared the DMM rBMSC-lacZ group. Latter was decreased compared to the DMM PBS group (Fig. 9B). There was a significant reduction of MIP1b in the rBMSC-SP treatment group and the PBS control only 2 weeks past sham surgery (suppl. Figure 8 A). After 16 weeks, MCP1 level was elevated in the sham-rBMSC-lacZ group compared to the sham-PBS group (Fig. 9C). At the 2 weeks time point, MCP-1 was lower in the sham-rBMSC-SP group than the sham-rBMSC-lacZ treated group (suppl. Figure 8B).

When looking at the three time points, 2, 8, 16 weeks after surgery, it is striking, that the concentration of some analytes - which are IL6, IL4, MCP-1, MIP1a, MIP1b and VEGFα- were increasing over the time in the rBMSC-SP treatment group after DMM surgery (suppl. Figure 8 C). For the rBMCS-αCGRP treated DMM groups, there was no increase over the three time points, but they took on a bell-shaped curve with a peak at week 8 for IL6, leptin, MCP-1, MIP1a, MIP1b and RANKL and a decrease again at 16 weeks (suppl. Figure 8D).

## Discussion

### Treatment models

To gain new insights into the pathophysiology of osteoarthritic joint damage and pain, we aimed to elucidate the role of the nociceptive neuropeptides SP and αCGRP, in the development and pathology of OA. We observed in in vitro studies a balance between a more catabolic effect of SP and an anabolic effect of αCGRP in healthy primary knee chondrocytes. We proposed that this balance is disturbed in chondrocytes from knee OA patients, since the catabolic effect of both, SP and αCGRP became predominant [7]. After induction of OA via DMM surgery in SP- and αCGRP- deficient mice, we demonstrated that the absence of both neuropeptides lead to age- and OA-related subchondral bone structural changes [13]. Thus, we were interested in the impact of supraphysiological SP and αCGRP levels in the knee joints on the development and progression of post-traumatic OA. We therefore i.a. injected modified rBMSC, which stably express and secrete the murine SP or αCGRP peptides, to achieve a continuous stimulation of the joint tissues with the neuropeptides during DMM-induced OA development. Recombinant neuropeptides have a short half-life and would have demanded to be injected several times a week to secure availability. By using rBMSC as delivery vehicles, we expected to bypass this limitation and are able to study mid/long-term effects of increased SP and αCGRP levels on knee joint pathology. MSCs in general have low immunogenicity (not expressing class II histocompatibility molecules) and also immune-modulatory capabilities [27]. That property may account for differences in the deterioration of the cartilage matrix of the medial and lateral tibia plateaus and the lateral femoral condyle between the PBS- and the rBMSC-lacZ control groups. Nano-CT analysis of the epiphyseal and the sub-articular morphology revealed reduced BV/TV in the rBMSC-lacZ group compared to the PBS group. Whether this is a direct effect of the i.a. applied treatment or if the bone changes are rather secondary due to changes in bone loading remains to be addressed. Accordingly, we suggest that some of the observed anabolic and analgesic effects could be assigned to the rBMSCs or their secretome independent from the genetic modification. Moreover, it should be noted that SP and αCGRP were produced endogenously in low amounts by naïve rBMSC adding to the effects of the neuropeptide transduction. Transduction of rBMSC with either the murine cDNA for SP or αCGRP, resulted in a high mRNA expression level but comparable low protein synthesis of the neuropeptides. The correlation between expression levels of protein and mRNA in mammals is relatively low, with a Pearson correlation coefficient of ~ 0.40 [28, 29]. Thus, we suggest post-transcriptional regulation as one potential reason for a strong induction of gene expression but a relatively low protein synthesis and corresponding secretion.

### Detection of primed rBMSC

We were unable to proof the presence of rat DNA in mouse knee sections from the treatment groups 1 week after i.a. application of the primed rBMSC. Either the cells were dead or emigrated into tissues outside of the joints or into the vasculature. We rather assume emigration as we would expect to still find rat DNA expelled by dying cells after a few days.

### OARSI scoring

Cartilage histomorphometry revealed, representative for the medial femoral condyle and the medial tibia plateau, that the induction of post-traumatic (PT) OA by DMM surgery was successful for all treatment groups with increasingly differing OARSI scores between DMM versus sham surgery over the experimental timeline. However, no significant differences between treatment groups in the medial compartments (tibia plateau/femoral condyles), except at 2 weeks for the rBMSC-lacZ versus PBS group, were detected. After 8 and 16 weeks, a clear and increasing score difference between sham and DMM animals in all treatment groups was observed in the medial compartments, whereas at the early 2 weeks time point only significant score differences between DMM and sham was observed in the rBMSC-lacZ and PBS (both controls) groups. Contrary, no significant score increase in both neuropeptide treated DMM versus sham groups in the medial compartments were detectable. Thus, we assume that if there are neuropeptide effects on cartilage matrix degradation, it mainly manifests in early OA.

In the lateral femoral condyles at 8 weeks after DMM/sham surgery we noted a reduced OARSI score of the DMM rBMSC-αCGRP mice versus DMM PBS mice. Notably, the DMM rBMSC-lacZ group also had a lower OARSI score compared to the DMM PBS group, suggesting that the presence of control rBMSC themselves also contributed to this decreased OARSI score in the lateral femoral condyles. In general, the OARSI scores remained low – indicating mild cartilage damage only - in the lateral joint compartments and no differences were found between DMM and sham groups at 2 and 8 weeks except for the lateral tibia plateau at 8 weeks for the PBS and rBMSC-SP groups. This observation supports an attenuation of cartilage matrix structural degradation via the presence of αCGRP and/or the rBMSC.

Overall, after application of supraphysiological SP or αCGRP we cannot report similar clear cut effects on medial cartilage damage as reported for the neuropeptide knockout animal study by our group after DMM-induced OA induction [13].

### IT-AFM analysis of cartilage biomechanical properties

Structural and biomechanical properties of joint tissues, i.e. cartilage, at a nanoscale level can be assessed by indentation-type atomic force microscopy (IT-AFM) [30, 31]. At 8 weeks after DMM surgery we observed an increase in matrix stiffness of middle zone (MZ) cartilage compared to the sham surgery groups indicating the beginning of OA-related cartilage matrix alteration. Effects on biomechanical cartilage properties after DMM/sham surgery were increased stiffness (shift of the curve to the right) of MZ cartilage in the three DMM-rBMSC treatment groups (lacZ, SP and αCGRP) compared to the DMM PBS group. This suggests that the injected rBMSCs have decisive rather a catabolic effect on the cartilage biomechanical properties after DMM and less pronounced after sham surgery, possibly indicating an early proteoglycan loss.

The other cartilage zones, superficial zone (SZ) and deep zone (DZ), revealed similar tendencies, but the MZ displayed the clearest results. The SZ constitutes the cartilage surface interface with the synovial fluid and is therefore the most exposed zone to (aberrant) mechanical load as induced by the DMM method, and thus is very sensitive to changes and stronger affected than the other zones, especially in the DMM groups where already cartilage deterioration can be assumed. Atypical bimodal distributions of the curves suggest that the matrix structure is about to be destroyed eventually resulting in fibrillations observed in late stages of OA.

The DZ is the most protected zone and presumably showed therefore the least effects of the surgeries and/or injections. The presence of both neuropeptides seemed to have an overall (light) preventing or reversing/softening effect compared to the rBMSC-lacZ injection in all cartilage zones and surgery models, with rBMSC-αCGRP having the most pronounced effect. In a previous study of our group, AFM identified a strong superficial zone (SZ) cartilage phenotype in Tac1−/− (no SP) sham mice [13]. Opposed to WT and αCGRP−/− mice, SZ cartilage of Tac1−/− mice softened 2 weeks after OA induction. MZ cartilage matrix stiffness increased 2 weeks after DMM surgery in all genotypes and remained higher even after 8 weeks in the neuropeptide deficient DMM mice. Thus, cartilage matrix stiffness showed more severe alterations in SP- and αCGRP-deficient DMM mice, in comparison to rBMSC-SP or rBMSC-αCGRP treated DMM mice in this study. The effects in knockout mice were both, OA-dependent and specific to neuropeptide deficiency (in sham mice without OA induction), whereas our i.a. injection model showed mostly OA- and rBMSC-dependent effects, with only minor impact of additional SP or αCGRP on cartilage stiffness. Either the 8 weeks time point was too early to observe differences in cartilage matrix mechanical properties between the treatments at the nanoscale level or the increase in concentration of the neuropeptides was too subtle to induce strong structural effects on cartilage matrix macromolecules.

### Mobility and activity of mice

Evaluation of the mobility revealed that at 8 weeks post surgery the DMM-rBMSC-αCGRP treatment group was more active than DMM-rBMSC-lacZ and-PBS treatment groups, whereas no difference between the DMM-rBMSC-SP group and both control groups was observed. Thus, we suggest a beneficial effect of αCGRP on the general mouse activity after DMM and possibly an analgesic effect on OA-mediated knee pain in those animals. At first glance, this appears to be in conflict with a study performed by Benschop and colleagues, where a neutralizing antibody to αCGRP showed promising efficacy in treating OA-related pain in a pre-clinical OA pain model [32]. A likely reason may be the usage of a different non-surgical rather inflammatory OA model in rats in this study where OA was induced by i.a. injection of monoiodoacetate, which provokes a strong inflammatory based pain sensation and is mostly used for analysis of drug effects on pain. As the αCGRP effect in our study was only detectable 8 weeks after OA induction, but no longer after 16 weeks (even though the rBMSC-αCGRP injection was repeated 2 times before the 16 week analysis time point), one can speculate that the effect of αCGRP is dependent on the OA stage, and may be lost during disease progression. Moreover, at the late time point of 16 weeks, a higher movement activity in rBMSC-αCGRP and -lacZ sham groups compared to the rBMSC-SP sham group was observed, indicating a possible unfavorable effect of supraphysiological SP concentrations in the joint independent of OA induction. It is noteworthy, that increased SP levels were found in the synovial fluid obtained from OA patients who underwent total knee replacement surgery, without knowing if increased SP concentration promoted OA progression or the other way around [33].

### Nano CT analysis of subchondral bone

The results of the nano-CT analysis in this study provided insights into the effects of rBMSC-αCGRP and rBMSC-SP treatments on bone microstructure. The recurring theme seen in the results suggests an anabolic effect of rBMSC-αCGRP at the later time points (16 weeks), which is even more pronounced when combined with the effect of injury (DMM surgery). On the other hand, treatment with rBMSC-SP had a rather catabolic influence, leading to adverse effects in bone at earlier time points (8 weeks), which again became more predominant after injury.

In the medial epiphysis, the reduction in bone volume fraction (BV/TV) observed in the 16-week DMM-rBMSC-αCGRP group compared to its PBS counterpart indicates a protective influence of rBMSC-αCGRP, counteracting the trauma-induced sclerotic response. The lower BV/TV in this group, at levels comparable to PBS sham controls, suggests preservation of bone structure, which may indicate a protective effect against OA-induced bone sclerosis [34].

Further support for the anabolic properties of rBMSC-αCGRP is seen in the subarticular bone morphometry, where lower BV/TV and trabecular thickness (Tb.Th) were detected in the 16-week DMM-rBMSC-αCGRP group compared to the corresponding PBS control. This indicates that rBMSC-αCGRP treatment is beneficial to maintain the micro-architectural integrity of bone in the face of OA progression, especially in the subarticular region. This corresponds with our prior research, where we illustrated the significance of αCGRP as a vital trophic factor with bone-promoting properties involved primarily in maintaining bone health and, to a lesser extent, in regulating cartilage stability [13]. Additionally, the findings are in line with a study by Schwab et al., which observed frequent and dense innervation of subchondral bone by nerve fibers expressing αCGRP, while those positive for SP were seldom found [35].

Interestingly, the reduced subchondral bone plate thickness at 16 weeks in both the sham and DMM groups treated with rBMSC-αCGRP suggests a paradigm where remodeling sclerotic processes typically associated with degenerative changes in the joint were alleviated. While a thinner subchondral bone plate is often associated with early OA [36–38], in this context it may represent a protective mechanism by preventing excessive bone thickening characteristic of more advanced stages of OA [39].

Conversely, the results suggest that rBMSC-SP treatment exerts a more catabolic effect, with adverse consequences for bone health, particularly at earlier time points (8 weeks) and exacerbated after DMM surgery. This trend of bone deterioration in the presence of rBMSC-SP is evident in several aspects of the analysis, and corroborates previous studies that have shown the presence of SP elevates resorption activities both in vitro and in vivo [8, 40].

The formation of irregular lateral osteophytes, which was predominantly observed in the DMM-rBMSC-SP groups, highlights the catabolic influence of this treatment. Osteophytes are often associated with more severe OA, and their development suggests a more advanced stage of disease or a more severe pathological response [41, 42].

In addition, the increased thickness of the medial subchondral bone plate in the 8-week DMM rBMSC-SP group compared to the PBS control mice indicates a negative effect of SP on the subchondral bone architecture. This thickening may contribute to increased stiffness and decreased joint mobility, both of which are adverse effects associated with OA progression [43]. In the subarticular bone analysis, the higher BV/TV in the rBMSC-SP group further underscored a sclerotic response, indicating an unfavorable impact on bone density and structure. The increase in calcified cartilage (CC) thickness in the 16-week sham-rBMSC-SP group, which is the only treatment-specific finding in CC thickness, indicates a catabolic effect independent of OA induction. In relation to OA, the deterioration of CC integrity reflects the severity of osteoarthritis, while CC thickening may impair joint function and contribute to the progression of OA pathology [44].

### Serum factors

Adiponectin, an adipocyte-derived hormone with multiple biological functions, is traditionally considered as an anti-inflammatory adipokine in various disease states. Several SNPs of the adipokine genes have been associated with knee OA but the results are inconsistent [45]. Therefore, the role of adiponectin in the pathogenesis of OA remains controversial. On one hand, adiponectin was found to play a pro-inflammatory role in OA, stimulating the expression of IL-6 and MMP-1/-3/-13 [46]. On the other hand, serum adiponectin levels were negatively associated with knee OA and synovial inflammation in DMM- and tibial fracture models [47, 48]. The highest level of adiponectin in our study was detected in the serum of rBMSC-αCGRP treated mice 16 weeks post-OA induction with a significantly increased adiponectin level compared to the DMM-rBMSC-lacZ und DMM-rBMSC-SP groups. This is of interest as in αCGRP-deficient mice adiponectin gene expression in white adipose tissue was increased and αCGRP-deficient mice were protected from high-fat diet-induced obesity suggesting that endogenous αCGRP is a key regulator of metabolism and energy homeostasis [49]. We did not observe any relevant weight changes in the rBMSC-αCGRP mice compared to the other treatment groups, presumably due to an initial mostly local and not systemic change of αCGRP level in the knee.

Hülser et al. assumed that systemic adiponectin induction is dependent on the stage of OA progression and is not reflected in local adiponectin expression [50]. As we did not detect a simultaneous systemic increase of other pro- inflammatory markers like IL1ß or IL17A together with the adiponectin level during OA progression in the serum of DMM-animals in our study, we suggest that adiponectin exerts rather an anti-inflammatory response in OA joints.

Su et al. found a causal relationship between a high circulating level of macrophage inflammatory protein-1 beta (MIP-1β/CCL4) and a decreased risk of OA [51]. In contrast, others reported that MIP-1ß (CCL4) and other members of the CC family of chemokines, are upregulated after joint injury and OA [52], and CCL3, CCL4, and CCL5 are known to be among the most highly upregulated gene products in human chondrocytes in response to IL-1β [53]. It is described that SP stimulates expression of CC chemokines and their receptors in neutrophils [54]. We detected an increased level of MIP-1ß in DMM rBMSC-SP treated mice serum in comparison to the DMM-rBMSC-lacZ group, supporting a catabolic role of SP.

Notably, it remains to be elucidated how i.a. injected neuropeptide primed rBMSC modulate concentration of cytokines/chemokines/growth factors in serum. We assume that–according to our negative PCR results in knee joint sections- rBMSC escaped from the joint into the lymphatic system or the blood circulation and from there into other body tissues, i.e. adipose tissue or bone marrow.

## Conclusion

We achieved a robust induction of OA by DMM surgery according to the OARSI scores from week 8 on in the medial cartilage compartments without substantial effects of the treatments. In the lateral compartments, OA induction was mostly observed in the lacZ- and PBS treatment groups at 16 weeks but less in the SP- and αCGRP groups indicating a location dependent (lateral only) OA attenuating effect of the neuropeptides.

IT-AFM analysis of the articular cartilage matrix in the MZ delivered the clearest results regarding surgery type and treatment compared to the SZ and DZ. The DZ showed only subtle effects due to its protected location inside the tissue and the SZ displayed disproportionally strong changes due to its sensitive location at the cartilage surface. The MZ revealed effects of the treatments on the matrix stiffness within each surgery model. The injection of rBMSC-lacZ had a clear stiffening effect on the cartilage matrix indicating a more severe/progressed OA development as in the other rBMSC treatment groups. The rBMSC-αCGRP and -SP treatment groups showed a slightly reduced matrix stiffening, suggesting that the presence of both neuropeptides had a softening effect on cartilage tissue delaying OA progression. Among the neuropeptide groups, αCGRP treatment induced a stronger softening effect and thus the higher protective influence on OA severity. However, the reduction in stiffness due to the presence of αCGRP and SP did not fully rescue the stiffening effect of the rBMSC-lacZ treatment.

Notably, we suggest a beneficial effect of rBMSC- αCGRP treatment on the general mobility and activity of the mice in our DMM model and possibly an analgesic effect on OA-mediated knee pain whereas rBMSC-SP treatment seems to reduce the mobility independent of OA induction suggesting a catabolic and/or pain related effect of SP.

Nano-CT analysis of bone microstructure provides compelling evidence that rBMSC-αCGRP treatment appears to have an anabolic effect on bone with potential protective properties against OA-induced bone loss. rBMSC-αCGRP treatment seems beneficial by maintaining the microarchitectural integrity of bone despite OA progression, especially in the subarticular region. In contrast, rBMSC-SP treatment appears to exert a more catabolic influence, leading to adverse bone changes, particularly at earlier time points and after DMM surgery. This extends to the formation of irregular lateral osteophytes, which was predominantly observed in the DMM-rBMSC-SP groups, an increase in calcified cartilage thickness, and a sclerotic response of the subarticular bone.

When analyzing serum factors, differences between treatment groups were clearly detected. Treatment with rBMSC-αCGRP after DMM surgery resulted in a significant increase of serum adiponectin compared to the SP- and lacZ groups. In contrast, serum level of pro-inflammatory MIP-1ß was increased in the SP-group compared to both control groups. Taking it together with the other serum factors, one can assume an anti-inflammatory, anabolic effect of adiponectin induced by αCGRP versus a catabolic effect of MIP-1ß induced by SP. How local changes of neuropeptide concentration in the knee joint affect systemic serum factors remains to be analysed.

All together, we suggest a general anabolic and protective effect of αCGRP on knee joint tissues whereas SP effects are more catabolic and destructive.

## Electronic supplementary material

Below is the link to the electronic supplementary material.

Supplementary Material 1

Supplementary Material 2

Supplementary Material 3

Supplementary Material 4

Supplementary Material 5

Supplementary Material 6

Supplementary Material 7

Supplementary Material 8

Supplementary Material 9

## Data Availability

The datasets used and/or analysed during the current study are available from the corresponding author on reasonable request.

## Declarations

### Usage of AI

The authors declare that they have not used Artificial Intelligence in this study.
