# Supplementary figures and images for: Effects of intra-articular applied rat BMSCs expressing alpha-calcitonin gene-related peptide or substance P on osteoarthritis pathogenesis in a murine surgical osteoarthritis model
Source: Stem Cell Res Ther. 2025 Mar 5;16:117. doi: 10.1186/s13287-025-04155-2 (PMC11884178; doi:10.1186/s13287-025-04155-2)

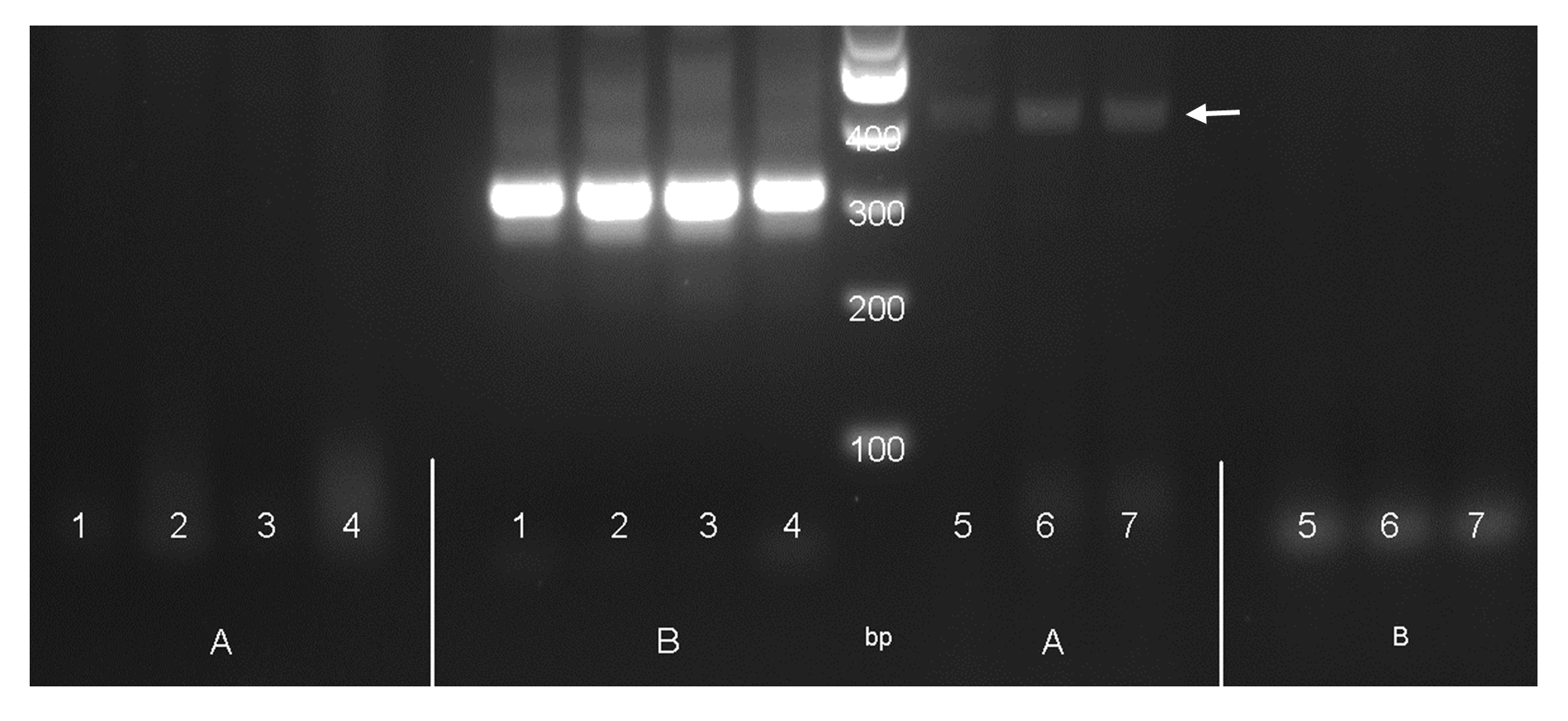

Supplement: Supplementary file 1 — Supplementary Material 1 [file 13287_2025_4155_MOESM1_ESM.tif]

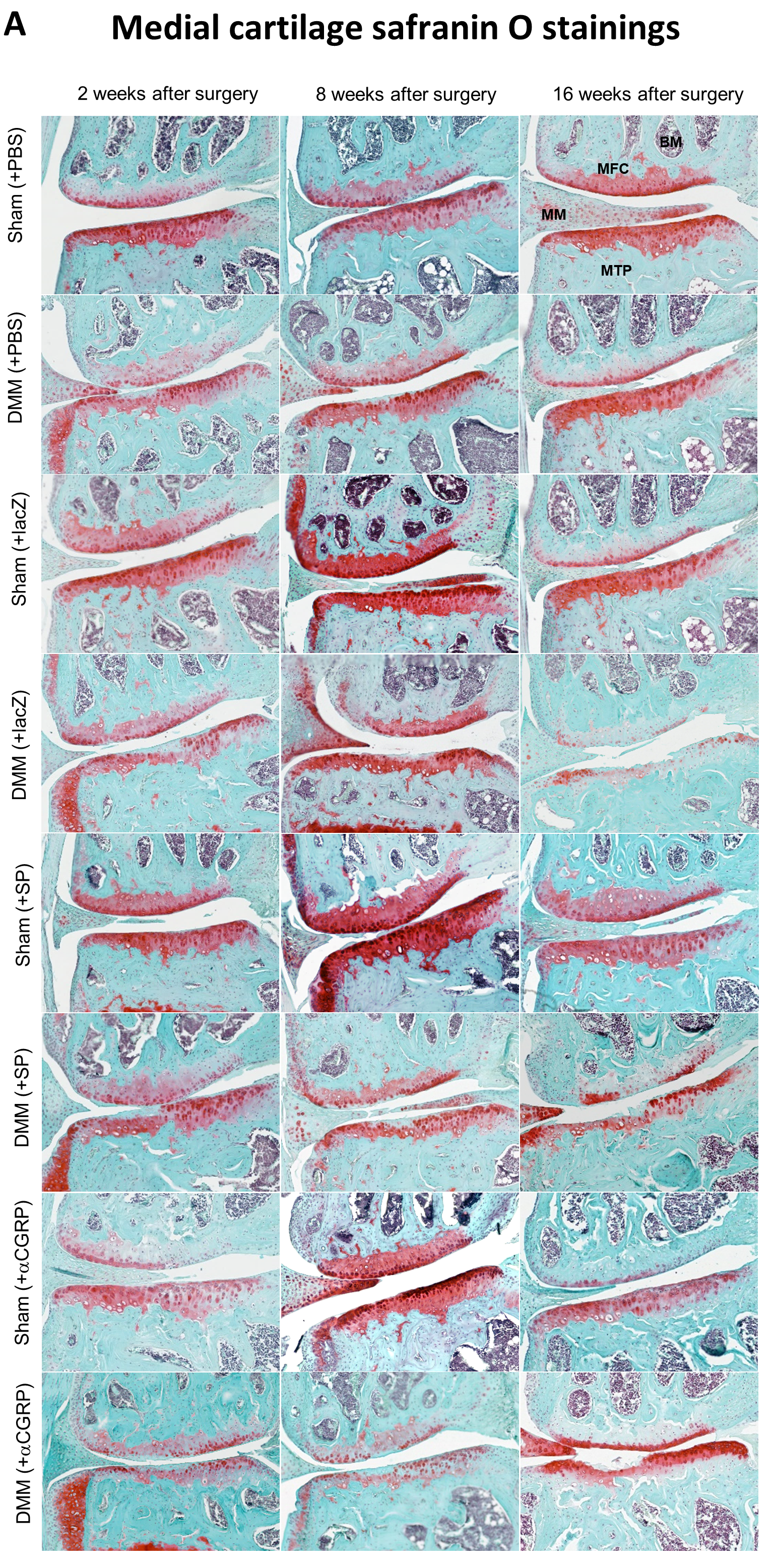

Supplement: Supplementary file 2 — Supplementary Material 2 [file 13287_2025_4155_MOESM2_ESM.png]

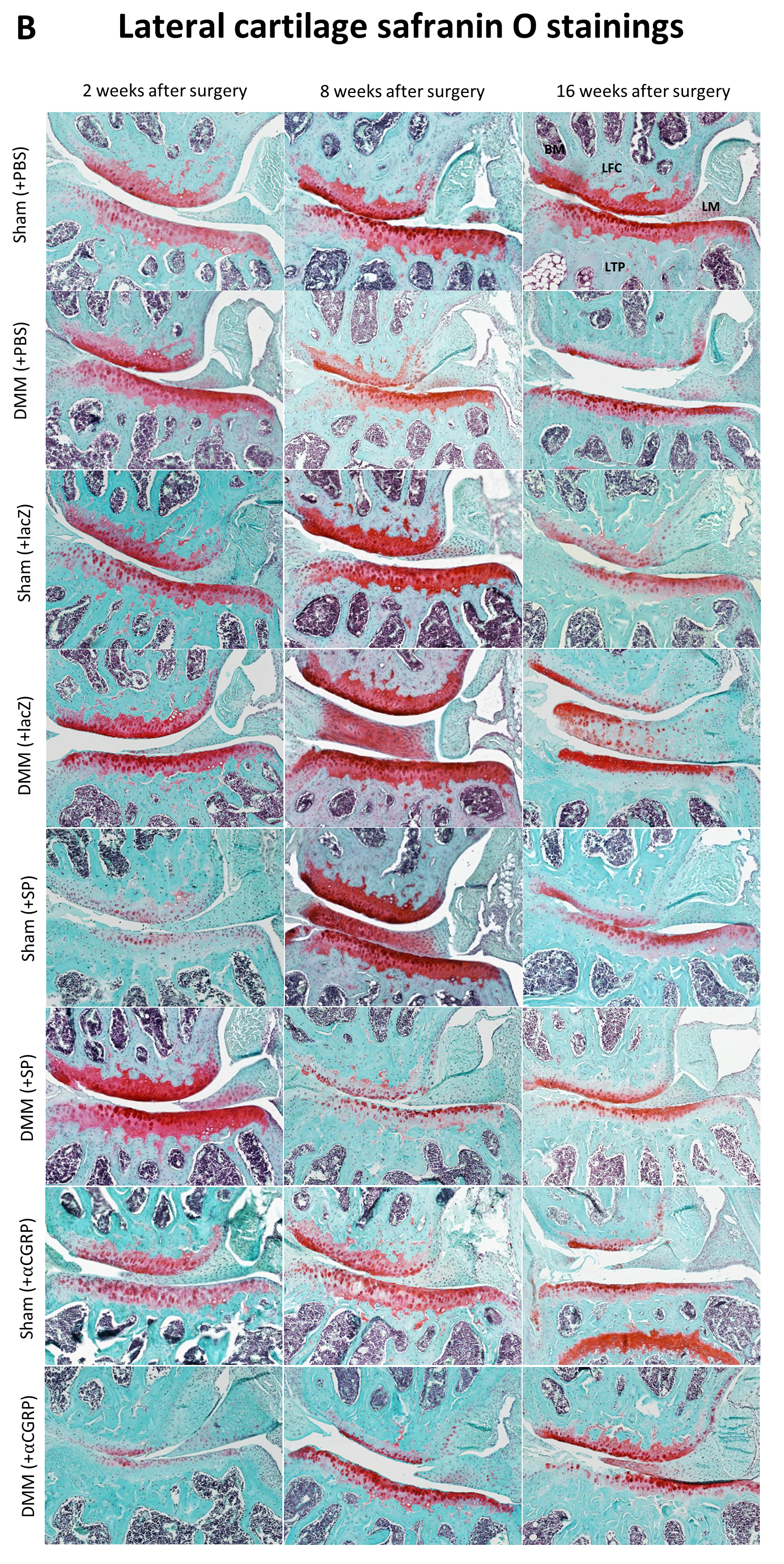

Supplement: Supplementary file 3 — Supplementary Material 3 [file 13287_2025_4155_MOESM3_ESM.png]

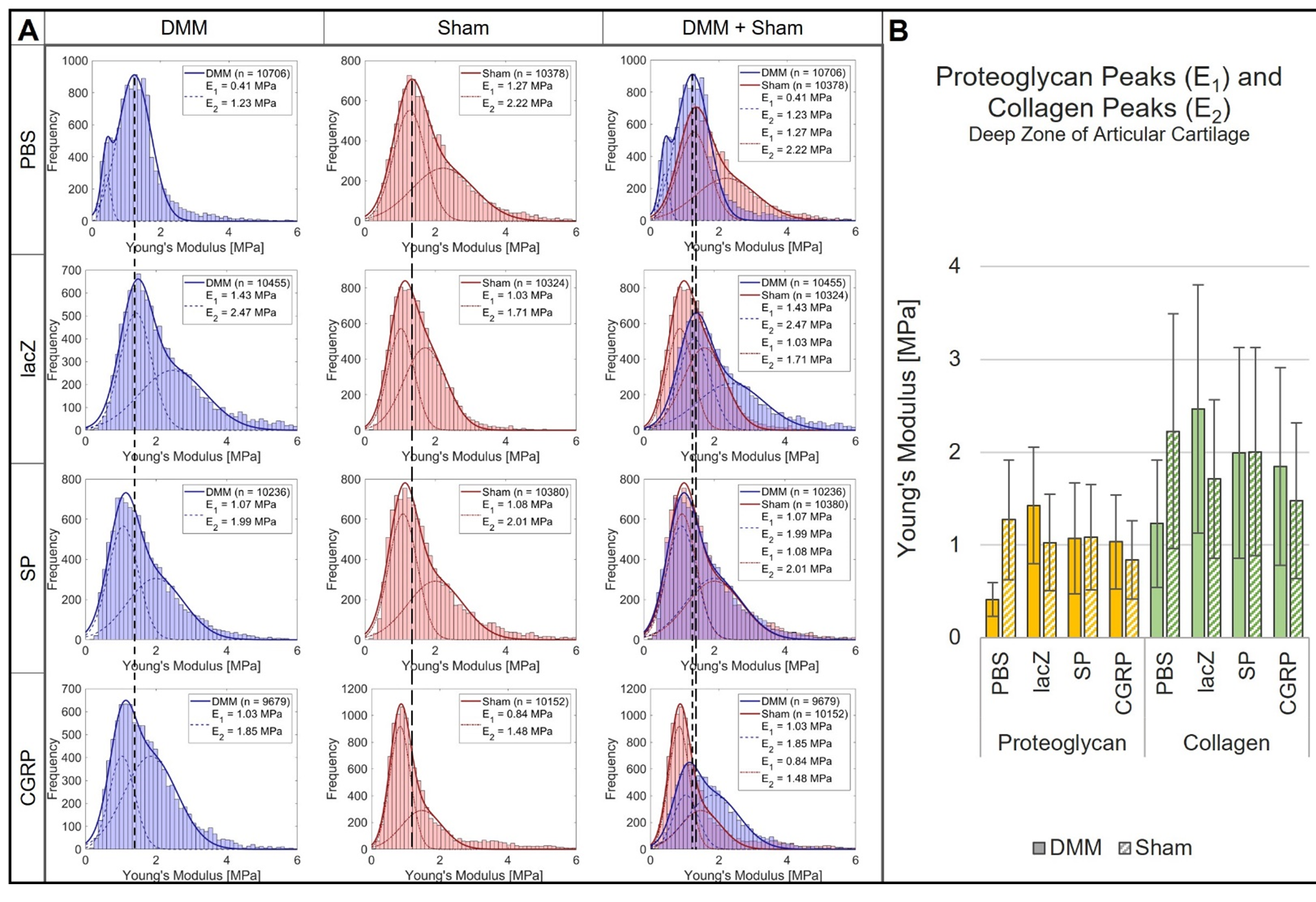

Supplement: Supplementary file 4 — Supplementary Material 4 [file 13287_2025_4155_MOESM4_ESM.tif]

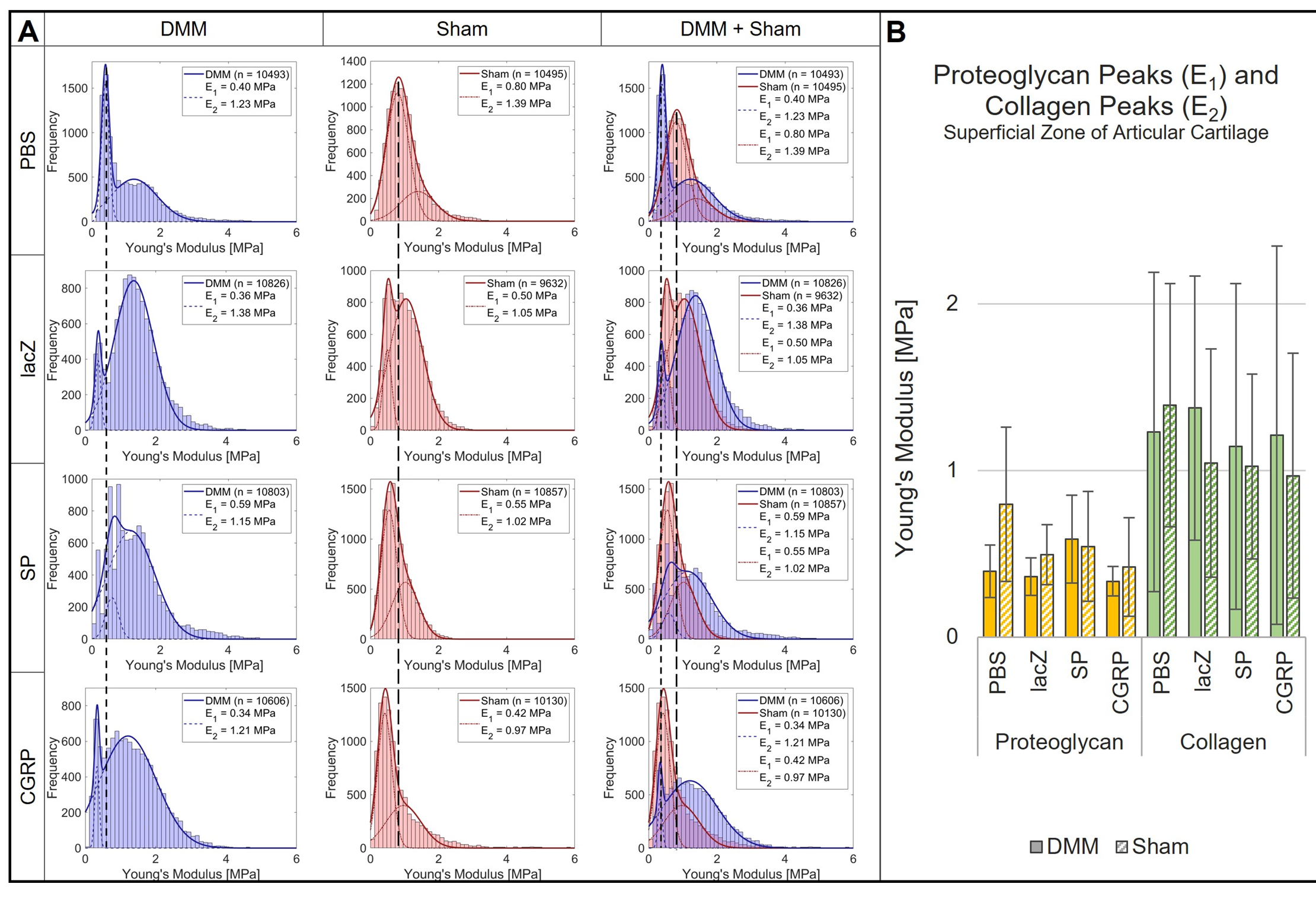

Supplement: Supplementary file 5 — Supplementary Material 5 [file 13287_2025_4155_MOESM5_ESM.tif]

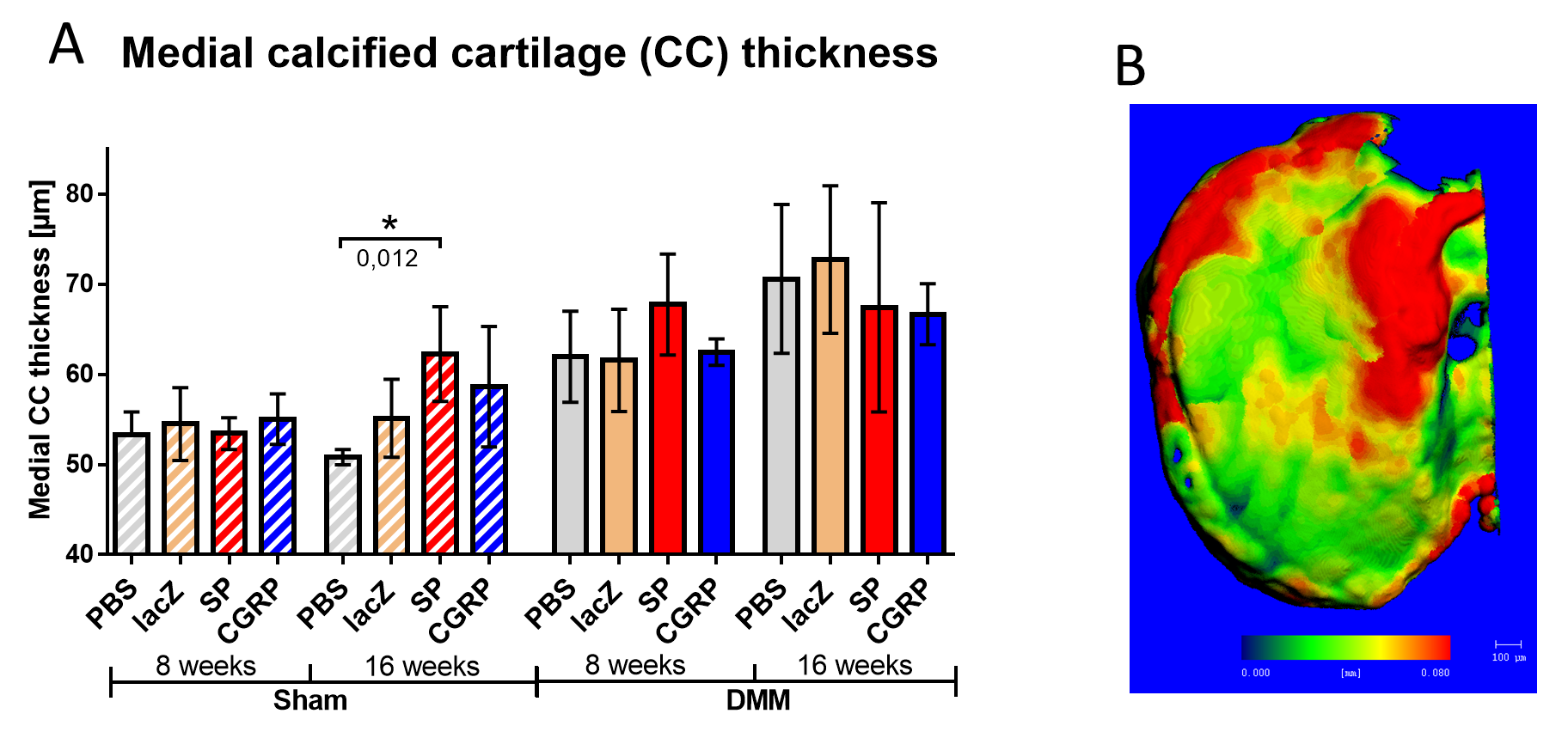

Supplement: Supplementary file 6 — Supplementary Material 6 [file 13287_2025_4155_MOESM6_ESM.tif]

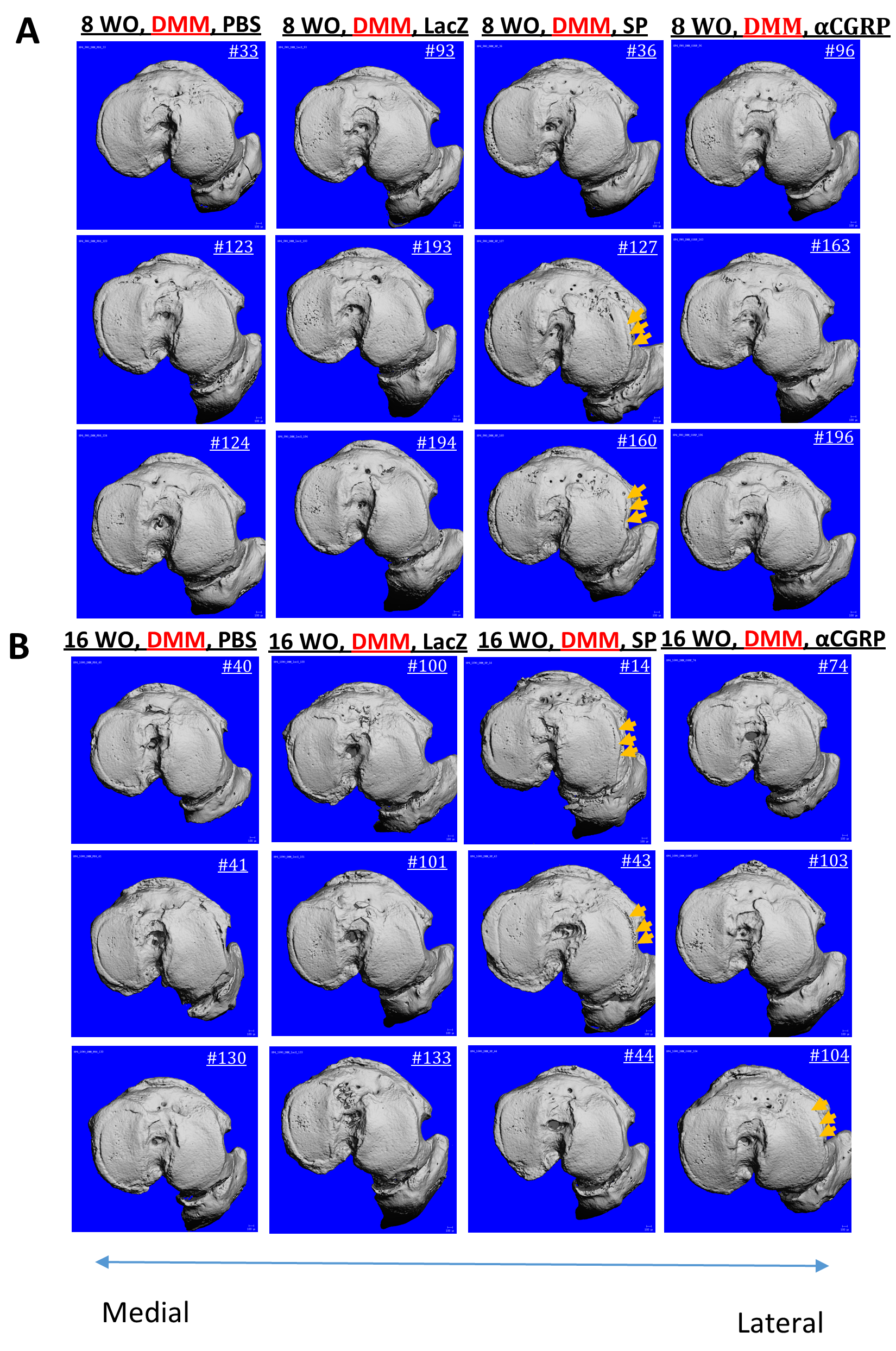

Supplement: Supplementary file 7 — Supplementary Material 7 [file 13287_2025_4155_MOESM7_ESM.tif]

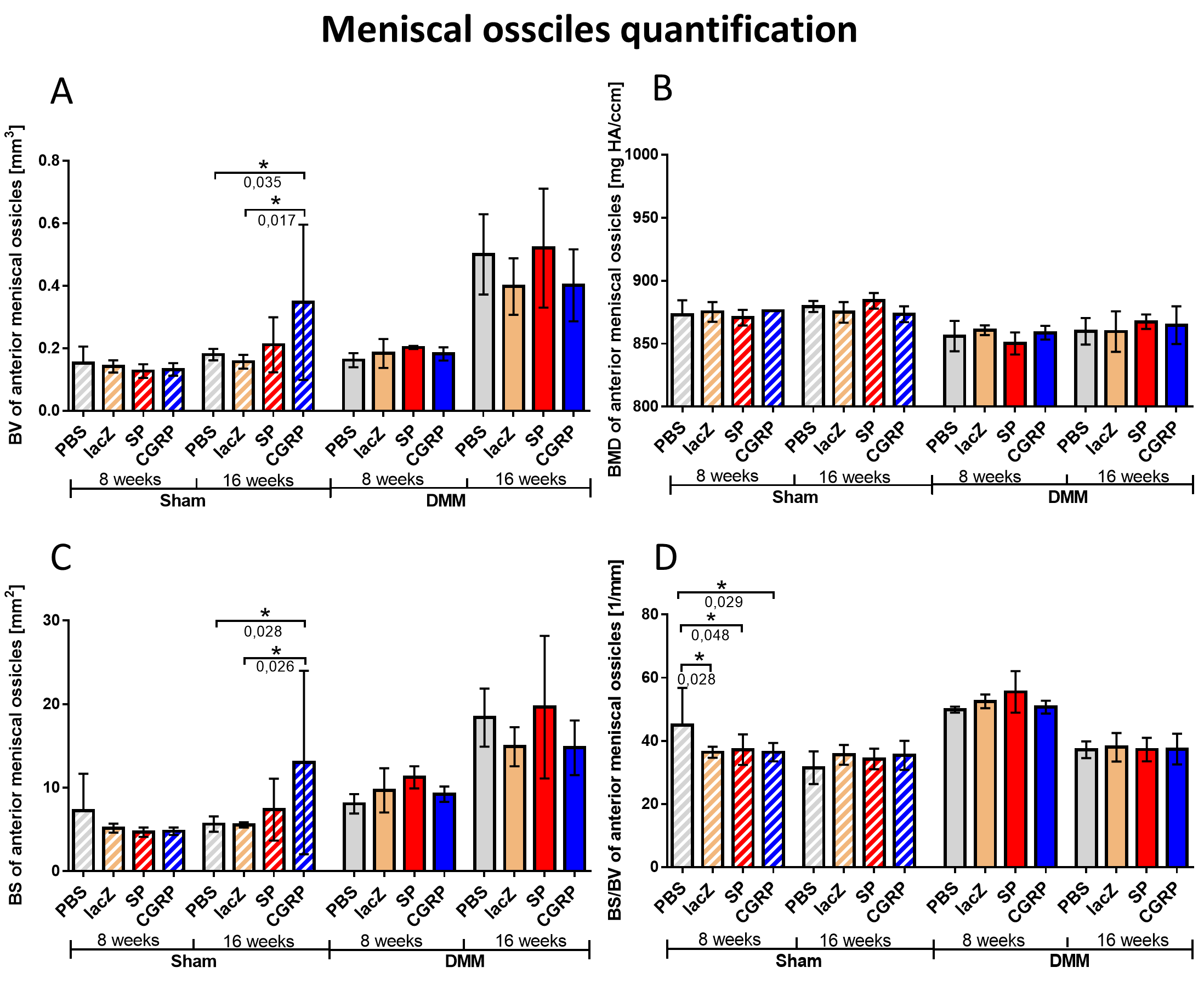

Supplement: Supplementary file 8 — Supplementary Material 8 [file 13287_2025_4155_MOESM8_ESM.tif]

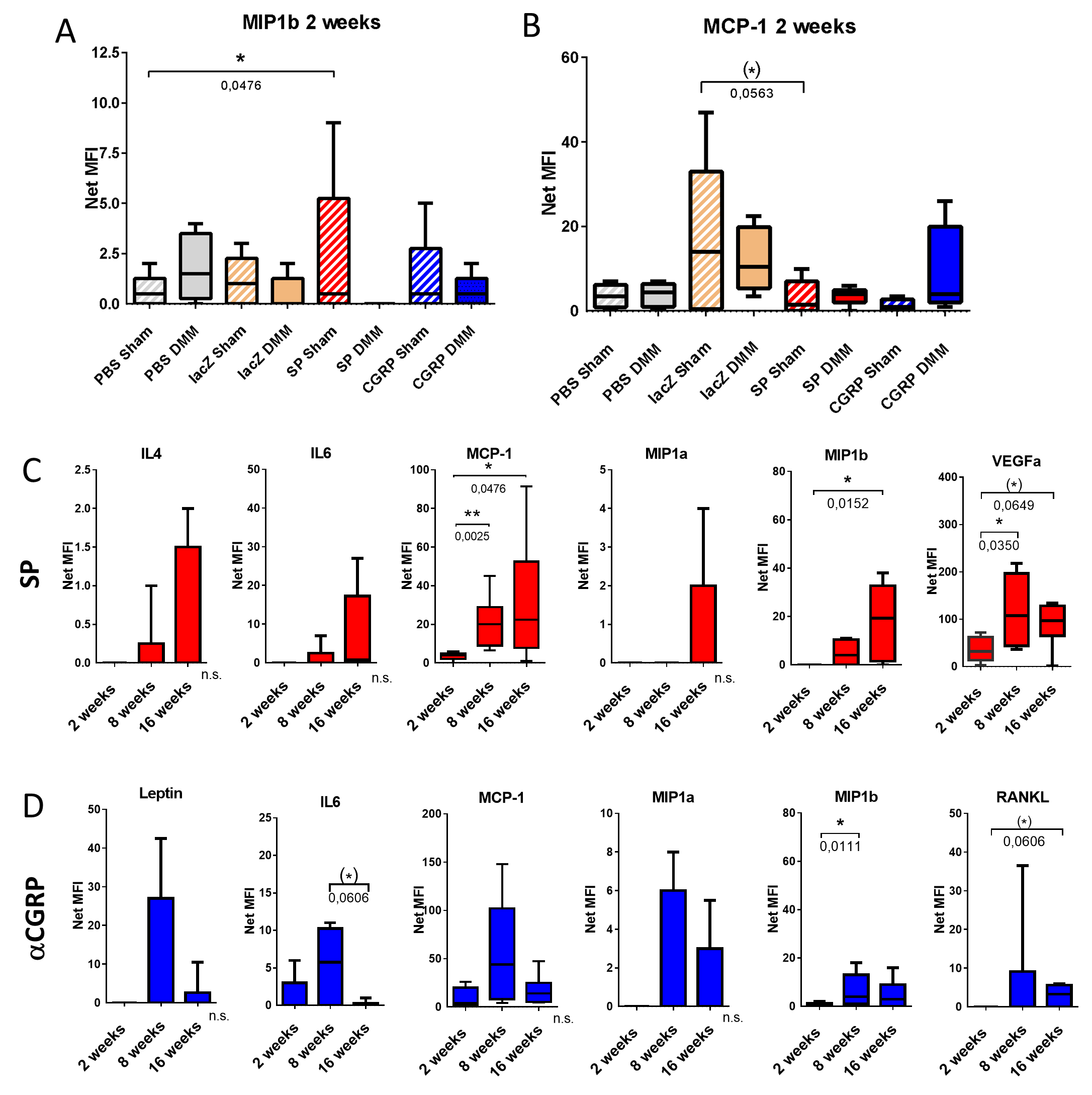

Supplement: Supplementary file 9 — Supplementary Material 9 [file 13287_2025_4155_MOESM9_ESM.tif]
